# Charlemagne's Summit Canal: An Early Medieval Hydro-Engineering Project for Passing the Central European Watershed

**DOI:** 10.1371/journal.pone.0108194

**Published:** 2014-09-24

**Authors:** Christoph Zielhofer, Eva Leitholdt, Lukas Werther, Andreas Stele, Jens Bussmann, Sven Linzen, Michael Schneider, Cornelius Meyer, Stefanie Berg-Hobohm, Peter Ettel

**Affiliations:** 1 Institute of Geography, Leipzig University, Leipzig, Germany; 2 Chair of Prehistory and Early History, Friedrich-Schiller University, Jena, Germany; 3 Institute of Geography, Osnabruck University, Osnabruck, Germany; 4 Leibniz Institute of Photonic Technology (IPHT), Jena, Germany; 5 Eastern Atlas, Berlin, Germany; 6 Bavarian State Department of Cultural Heritage, Munich, Germany; New York State Museum, United States of America

## Abstract

The Central European Watershed divides the Rhine-Main catchment and the Danube catchment. In the Early Medieval period, when ships were important means of transportation, Charlemagne decided to link both catchments by the construction of a canal connecting the Schwabian Rezat and the Altmühl rivers. The artificial waterway would provide a continuous inland navigation route from the North Sea to the Black Sea. The shortcut is known as Fossa Carolina and represents one of the most important Early Medieval engineering achievements in Europe. Despite the important geostrategic relevance of the construction it is not clarified whether the canal was actually used as a navigation waterway. We present new geophysical data and *in situ* findings from the trench fills that prove for the first time a total length of the constructed Carolingian canal of at least 2300 metres. We have evidence for a conceptual width of the artificial water course between 5 and 6 metres and a water depth of at least 60 to 80 cm. This allows a crossing way passage of Carolingian cargo scows with a payload of several tons. There is strong evidence for clayey to silty layers in the trench fills which reveal suspension load limited stillwater deposition and, therefore, the evidence of former Carolingian and post-Carolingian ponds. These findings are strongly supported by numerous sapropel layers within the trench fills. Our results presented in this study indicate an extraordinarily advanced construction level of the known course of the canal. Here, the excavated levels of Carolingian trench bottoms were generally sufficient for the efficient construction of stepped ponds and prove a final concept for a summit canal. We have evidence for the artificial Carolingian dislocation of the watershed and assume a sophisticated Early Medieval hydrological engineering concept for supplying the summit of the canal with adequate water.

## Introduction

### European Dimension of a Carolingian canal

Central Europe is covered by a dense network of navigable rivers. Along with overland routes they built the backbone of communication and commerce during the Middle Ages. Rivers formed the infrastructural link between traffic and economic systems of the Black Sea and Mediterranean basin on the one hand and of the North Sea and Baltic region on the other hand in this period [1]–[3]. Although generally disregarded in historical, archaeological and geoarchaeological research, ports and watersheds are the most important nodal points within this network [4].

**Figure 1:**
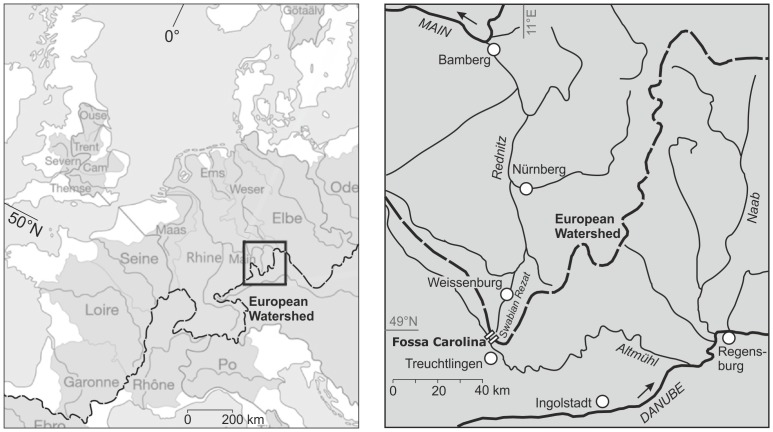
Fossa Carolina bridging the Central European Watershed. a) The outstanding role of the Fossa Carolina for Early Medieval shipping by bridging the Central European Watershed between the Rhine-Main and the Danube catchments. b) The Fossa Carolina was constructed by Charlemagne for linking the Swabian Rezat and the Altmühl Rivers. The figure is not similar to formerly published figures.

**Figure 2:**
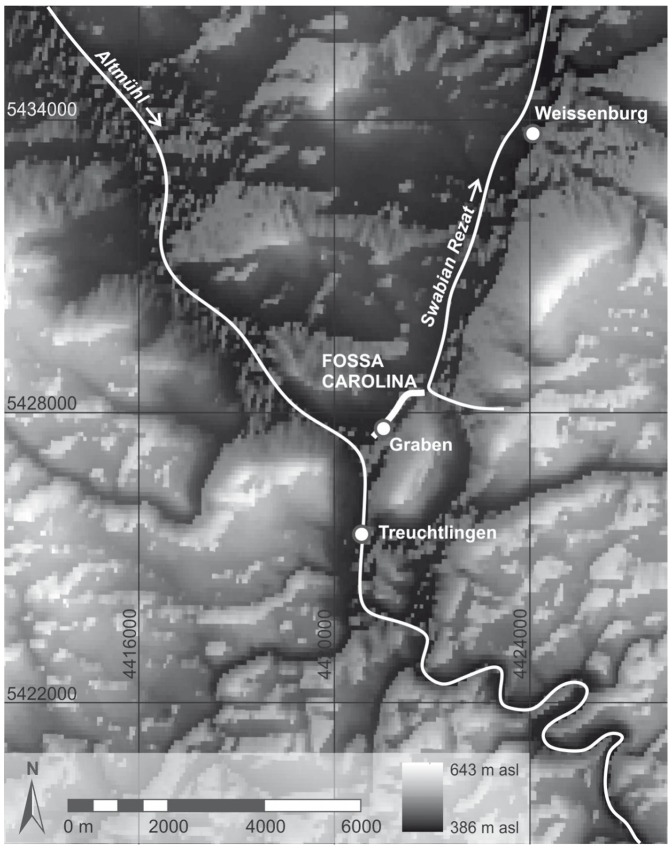
The local topographical setting of the Fossa Carolina. The canal is located across a valley watershed between the Altmühl River (Danube catchment) and the Swabian Rezat River (Rhine-Main catchment) within the Treuchtlingen valley junction at the piedmont of the Franconian Jura escarpment. Data source: Open access SRTM data (3 arc-second, 90 m raster, [72]) and OpenStreetMap [73].

During the Early to High Medieval period (cf. Table S1) the entire region between Denmark and Italy was controlled by powerful elites which were extraordinarily mobile, building up itinerant kingships and huge economic networks controlled by religious institutions [5]–[8]. Freund [9] highlights the important role of Central European river valleys for the communication networks of these groups. The basic work of Eckholdt [10] features methodological problems. Here, the role of the small rivers seems to be underrepresented [11]. However, until now there is poor knowledge about the location of inland ports, the explicit medieval navigability of the rivers and the bridging of watersheds between these rivers and their catchment areas [12]. So far there is mainly evidence for small and simple constructed medieval inland ports and hythes [13], [14]. But there is one extraordinary piece of engineering which highlights the enormous economic and human resources which have been mobilised for the building of navigable waterways: the Fossa Carolina which is the major focus of our geoarchaeological study presented here. The canal mirrors the first attempt to bridge the Central European watershed during the Early Medieval period [15]–[18]. The successful use of the canal would connect the Rhine-Main-catchment with the Danube catchment which means a continuous shipping lane between the Atlantic and the Black Sea (Fig. 1a and b, Fig. 2). Hence, a successful bridging of the Central European watershed would be a performance of engineering of European dimension in the scope of Early Medieval infrastructure and economies [1], [19]. The demand on economic resources for the canal construction was enormous, but even bigger was the potential advantage of controlling this pivotal point in the inland navigation network of Europe, connecting such tremendous powers as the Carolingian and the Byzantine Empires [1]. However, political control was a prerequisite for the canal construction and the outstanding man who gathered this control in a long series of conflicts and wars during the late 8^th^ century AD was Charlemagne [8], [20], [21].

### Historical tradition

Decisive information about Charlemagne's canal derives from Early Medieval written sources. The annals of the Frankish empire record for the year 793 AD a major construction site at the eastern edge of the realm: *in autumn Charlemagne was coming per ship from Ratisbon to the big canal between the Altmühl and the Rezat rivers […]* (Annales Regni Francorum, [22]). Numerous Carolingian sources describe the construction of the canal in contradictory details [23], [24]. In the Annals of Einhard the following description is found: *as the king […] was convinced that it might be possible to build a navigable canal between the Altmühl River and the Rezat River arriving suitable from the Danube River into the Rhine River he betakes himself to the place, assembles a large amount of workers and was staying the entire autumn at the construction site […] but without avail: due to ongoing rainstorms* *[…]* *it was not possible to achieve the progress*. *The material which was excavated by the workers during the day collapsed during the night* (Annales Regni Francorum, [22]). Legends about the construction of the Carolingian canal are mentioned in numerous more recent sources. This is the origin of the Ludwig-Danube-Main Canal which was dedicated in 1843 and the modern Rhine-Maine-Danube Canal which was finished in 1992 after 32 years of construction [25].

Different reasons hinder a clear interpretation of the written sources. Especially the real finishing of the construction and the effective use of the building as a waterway were contradictorily discussed [26]–[29]. But accomplished or not, Charlemagne's vision of the Fossa Carolina highlights the major importance of waterways for the transportation of people and goods in the supra-regional network of Early Medieval traffic and customs facilities [1], [5], [30], [31].

### Building remains and previous knowledge about use and engineering concept of the canal

**Figure 3:**
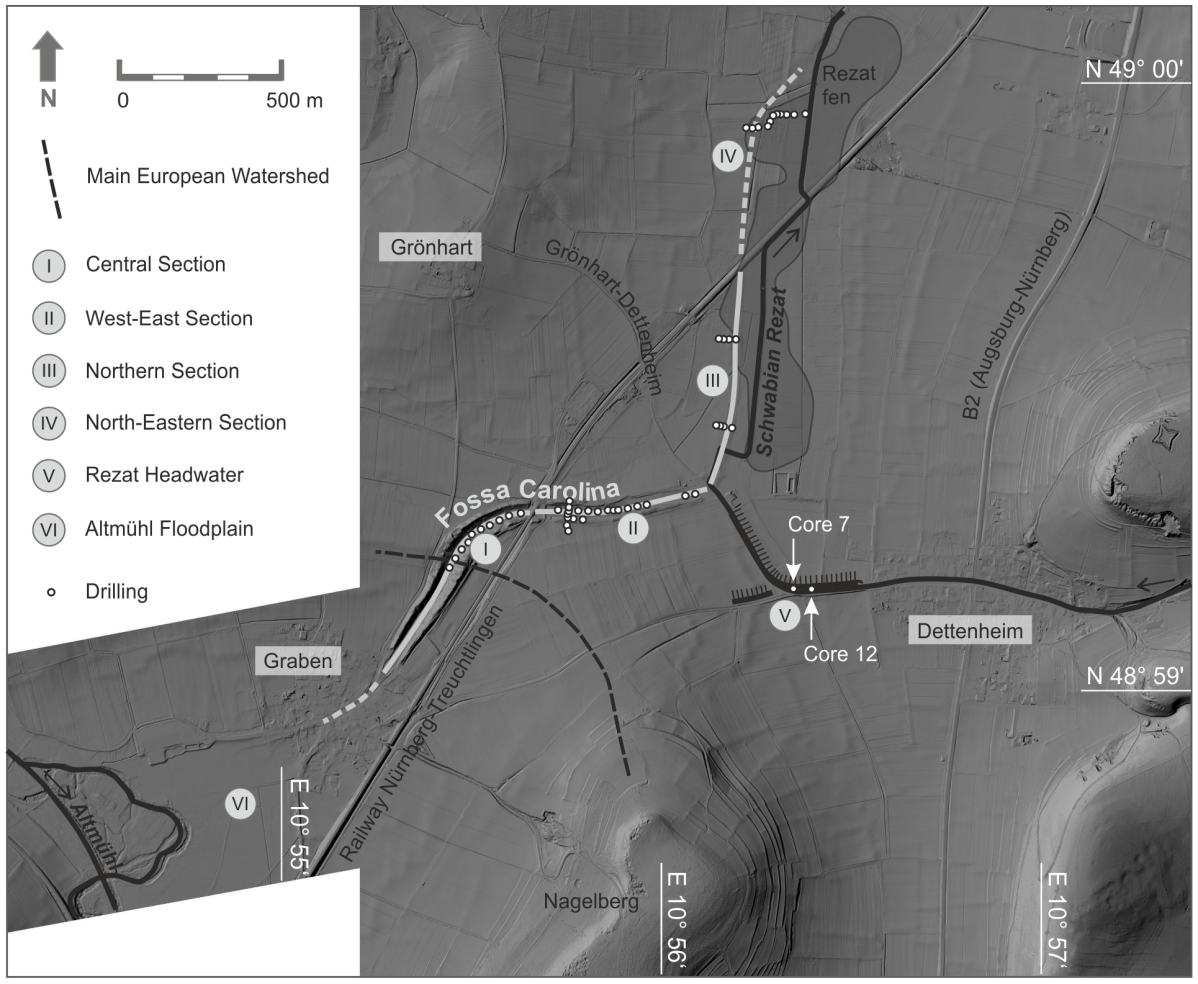
The course of the Fossa Carolina and the subdivision in six sections. I) Central Section, II) West-East Section, III) Northern Section, IV) North-Eastern Section, V) Rezat Headwater with cores 7 and 12 [35] within the former assumed dam for a Rezat reservoir [15], and VI) Altmühl Floodplain. For detailed drilling positions of the sections I, II, III, and IV see Figs 4, 8 and 12. Raw data source: LIDAR data are cordially provided by Bavarian Land Surveying Office for illustrative purposes.

At the northern edge of Graben village impressive remnants of the Fossa Carolina are visible (Fig. 3). A first topographic documentation of the canal by C. L. Thomas reveals a length of 1230 m, an average breadth of the canal of 30 m and banks with maximum heights of 6.5 m and maximum breadths of 40 m [32]. G. Hock reconstructed the Carolingian excavation level by a first archaeological survey in 1910 [32]. Based on these results and the available written sources Schwarz [32] concluded that the building was never completed. First aerial photographs from the 1970s and 1980s indicate that remnants of the former banks can be tracked at least 1000 m further north in the zone of the Rezat fen [27]. Regarding the southern continuation of the canal, Koch et al. [27] assumed from oral tradition and poorly documented former casting pits that the present-day Graben main street follows the former course of the Carolingian canal in the direction to the Altmühl River.

Koch et al. [27] and Koch and Leininger [33] conducted a first drilling campaign in the fills of the central part of the canal. Fifteen cores feature a peat layer and unfortunately poorly documented first palynological evidence for former ponds [34]. Regarding the levels of the drilled peat layers, Küster [34] assumed that the level of excavation of the trench does not reach the necessary depth for a continuous canal. Hence, the building was never finished or was already initially planned as a summit canal. This corresponds with the descriptions of the Carolingian Annales Guelferbytani. Here, it is mentioned for 793 AD that the boats were towed through the water and overland (Annales et chronica aevi Carolini, [24]). However, there is no clear geoarchaeological evidence for a planned summit canal yet. Survey works by Koch et al. [27] and Koch and Leininger [33] indicate that the West-East Section (Fig. 3) of the canal does not follow the natural Rezat drainage system to the north. Rather, the canal might be connected in the zone of a noticeable rectangular bend with the uppermost course of the slightly diverted Rezat stream to subjoin the canal with additional water. Here, Koch [15] assumed that the embankment of the road between Grönhart and Dettenheim (Section V, Fig. 3) belongs to remnants of a Carolingian reservoir which might be constructed for supplying the Fossa Carolina with water from the Rezat spring. However, new ^14^C data from *in situ* dating of the embankments (Fig. 3) reveal High Medieval ages (Tables 1 and 2, [35]) and give no evidence for a Carolingian dam. The lowermost strata of the embankment reveal an age of 1194–1246 cal AD, followed by a younger heightening during the 15^th^ century AD (Table 2, [35]).

**Table 1:** Radiocarbon ages of the Fossa Carolina trench fill (Part 1).

Mean depth [cm below surface/m a.s.l.]	Core	Location	Lab No.	Material	^14^C [BP]	Calibrated 1σ range [cal. AD]	δ^13^C (‰)	Reference (if already published)
375/411.8	A	Trench fill (peat layer)	KIA36403	Wood	973±28	1024–1116	−26.1	Leitholdt et al. 2012
460/410.9	A	Trench fill (sandy layer)	KIA36404	Charcoal *Quersus sp.*	1267±27	692–762	−23.7	Leitholdt et al. 2012
610/409.4	A	Trench fill (sandy layer)	KIA36406	Charcoal *Quercus sp.*	1269±27	691–759	−24.5	Leitholdt et al. 2012
245/414.2	G	Trench fill (peat layer)	SU-GL-21	Wood	805±30	1207–1251	−27.5	Leitholdt et al. 2014
368/413.0	G	Trench fill (peat layer)	SU-GL-22	Wood	1180±30	793–879	−27.6	Leitholdt et al. 2014
238/414.2	H	Trench fill (peat layer)	SU-GL-23	Wood	765±30	1228–1266	−30.6	Leitholdt et al. 2014
386/412.8	H	Trench fill (peat layer)	SU-GL-24	Wood	1150±30	827–939	−27.1	Leitholdt et al. 2014
265/414.1	I	Trench fill (peat layer)	SU-GL-25	Wood	870±30	1078–1200	−28.2	Leitholdt et al. 2014
185/414.8	J	Trench fill	SUERC-42068	Wood *Quercus sp.*	834±25	1178–1232	−26.6	
275/413.9	J	Trench fill (peat layer)	SUERC-42069	Wood *Quercus sp.*	934±23	1048–1136	−25.1	
165/415.2	K	Trench fill (peat layer)	SUERC-42070	Caulis	874±25	1079–1195	−27.1	
225/414.6	K	Trench fill (peat layer)	SUERC-42074	Caulis	942±25	1043–1133	−27.4	
335/413.5	K	Trench fill	SUERC-42075	Wood	1253±26	701–773	−29.4	
355/413.3	K	Trench fill	SUERC-42076	Wood	1163±26	813–923	−27.9	
410/412.9	K	Trench fill	SUERC-42140	Wood *Alnus sp.*	1170±37	797–921	−28.7	
282/414.5	L	Trench fill (peat layer)	SUERC-42141	Wood *Salix sp.*	806±37	1193–1251	−27.4	
375/413.6	L	Trench fill (peat layer)	SUERC-42142	Wood	860±37	1087–1211	−28.2	
465/412.6	L	Trench fill	SUERC-42143	Wood	1110±37	893–969	−27.0	
230/415.3	M	Trench fill (peat layer)	SUERC-42147	Wood *Salix sp.*	782±37	1216–1260	−26.1	
474/412.9	M	Trench fill (trench bottom)	SUERC-42148	Wood *Salix sp.*	1223±37	723–851	−27.0	

Calibration (one sigma) of the conventional ages was performed using the Cologne Radiocarbon Calibration Program (quickcal2007 ver.1.5, [48]). KIA samples were conducted at Kiel AMS facility. SU-GL and SUERC samples were conducted at Glasgow AMS facility. MAMS samples were conducted at Mannheim AMS facility.

**Table 2:** Radiocarbon ages of the Fossa Carolina trench fill (Part 2).

160/415.4	B	Trench fill (peat layer)	KIA36407	Caulis	307±22	1524–1624	−27.3	Leitholdt et al. 2012
300/414.0	B	Trench fill (peat layer)	KIA36408	Wood	882±29	1067–1189	−29.5	Leitholdt et al. 2012
390/413.1	B	Trench fill	KIA36409	Wood	859±29	1149–1207	−30.9	Leitholdt et al. 2012
256/414.9	O	Trench fill (sapropel layer)	SUERC-44081	Wood *Salix sp.*	713±29	1266–1286	−28.4	
550/412.0	Q	Trench fill	SUERC-44082	Wood *Quercus sp.*	1271±29	689–761	−26.4	
87/416.7	S	Trench fill (sandy layer)	MAMS 18371	Wood *Juniperus sp.*	316±18	1523–1619	−20.3	
541/412.1	S	Trench fill (trench bottom)	MAMS 17461	Wood *Quercus sp.*	1312±17	666–708	−22.5	
482/413.0	T	Trench fill (peat layer)	SUERC-44083	Wood *Salix sp.*	1156±29	819–933	−29.4	
285/414.9	W	Trench fill (peat layer)	SUERC-44085	Seed *Sparganium sp.*	1021±29	990–1020	−24.1	
341/414.3	W	Trench fill (peat layer)	SUERC-44084	Wood *Salix sp.*	1186±26	792–870	−28.0	
369/414.5	Z2	Trench fill (sapropel layer)	SUERC-44089	Wood *Salix sp.*	1101±26	903–971	−28.9	
485/413.7	Z3	Trench fill	SUERC-44090	Wood *Salix sp.*	1234±29	716–836	−28.1	
75/421.2	7	Embankment	KIA38898	Charcoal	330±35	1498–1614	−21.0	Berg-Hobohm and Kopecky-Hermanns 2012
85/421.1	7	Embankment	KIA38899	Charcoal	445±30	1430–1454	−27.5	Berg-Hobohm and Kopecky-Hermanns 2012
138/420.6	7	Embankment	KIA38900	Charcoal	815±30	1194–1246	−23.2	Berg-Hobohm and Kopecky-Hermanns 2012
189/420.0	7	Buried A horizon	KIA38901A	Charcoal	1485±40	543–611	−24.7	Berg-Hobohm and Kopecky-Hermanns 2012
189/420.0	7	Buried A horizon	KIA38901B	Humic acid	1990±40	−38–46	−23.4	Berg-Hobohm and Kopecky-Hermanns 2012
74/422.3	12	Embankment	KIA38902A	Charcoal	340±30	1493–1609	−22.6	Berg-Hobohm and Kopecky-Hermanns 2012
74/422.3	12	Embankment	KIA38902B	Humic acid	320±35	1506–1616	−21.7	Berg-Hobohm and Kopecky-Hermanns 2012
367/414.3	QP-1	Trench fill (trench bottom)	MAMS 18372	Charcoal	1338±17	656–670	−30.7	
137/415.6	407-1	Trench fill (peat layer)	MAMS 18374	Charcoal	410±16	1444–1464	−28.5	
155/413.7	256-3	Trench fill (trench bottom)	MAMS 18375	Wood	1158±18	829–927	−23.6	

During a first survey our research group drilled seven cores in the Central Section of the canal. Radiocarbon dating feature for the first time a chronological model for the trench fills [36]. Our findings show a clear stratigraphic order of the fills. The oldest fills include wood remnants of Carolingian age. Therefore, the former hypotheses of an initial Roman age of the canal [37] must be discarded.

### Aims of our study

In former times archaeological and historical research about the Fossa Carolina was only focused on a potential Carolingian use of the construction [15]. First ^14^C data from the canal fills [17], [36] but also from the potential dam of the reservoir [35] do not support this concept. Hence, we have to start thinking about an alternative water engineering concept. In this study we focus on the probable course of the canal from Graben village to the Rezat fen. The course includes the Central Section, the West-East Section, the Northern Section and the North-Eastern Section (Fig. 3).

- For the first time we want to obtain detailed chronostratigraphical data from the trench fills (Fig. 3), scrutinising the possible evidence for open water bodies and, therefore, the potential use of the canal for shipping.
- We want to improve the stratigraphical model for a better reconstruction of the trench refilling process. Up to now, it is not clear, if the Carolingian canal collapsed due to the abrupt re-deposition of the bank material into the excavated trenches.
- We generally want to improve the knowledge about the Carolingian progress of construction works, and we aim to clarify the advised minimum water depth of the completed canal getting an idea about minimum requirements for Early Medieval shipping routes. Here, multiple reconstructions of cross sections of the canal course are required.
- The Fossa Carolina is an outstanding example of Early Medieval hydraulic engineering know-how. This study aims to reconstruct the longitudinal profile of the Fossa Carolina including the levels of excavation and the potential evidence for the artificial dislocation of the watershed. Here, our focus is to clarify the probable concept of a summit canal.
- Regarding the European dimension of the project, the detection of the confluence of the Fossa Carolina with the Altmühl River (Section VI in Fig. 3) will be a challenging part of future geoarchaeological research. In this study, we want to obtain for the first time *in situ* evidence about the potential extension of the canal course in the northern direction. Assuming that there was a navigable connection between the canal and the Rezat River, we must find remnants of a canal in the North-Eastern Section (Fig. 3).

## Geographical setting

The Fossa Carolina is located in the Southern Franconian Jura. It is surrounded by Upper Jurassic (Malm) carbonate rocks and Middle Jurassic Aalenian (Dogger Beta) sandstones [38] (Fig. S1, Fig. 2). The Swabian Rezat River (Rhine-Main catchment) and the Upper Altmühl River (Danube catchment) flow through undulating Middle Jurassic Aalenian (Dogger Alpha) and Lower Jurassic (Lias) foothills. The Fossa Carolina is not directly situated on the clayey sediments of the Middle Jurassic Aalenian (Dogger Alpha), but rather on fine clayey remains of Miocene lake deposits and Quaternary fills. The Miocene lake resulted from a meteorite impact that sealed the Miocene drainage system [38]–[40]. In the Central and West-East Sections limnic Miocene clays are covered by Quaternary fills consisting of fluvial sands down to a depth of 5 m [38], [39]. The Northern and North-Eastern Sections show different geological preconditions to the Central and West-East Sections. These are characterized by a natural, relatively wide valley floor with only slightly inclined valley edges. The groundwater table is naturally very high resulting in the development of the Rezat fen (Fig. 3). The Rezat fen comprises a part of the upper course of the Rezat stream, which originates in a V-shaped valley in the East of Dettenheim. The V-shaped valley is incised in the water-bearing Middle Jurassic Aalenian sandstone formation of the Franconian escarpment [38].

The location of the Fossa Carolina is closely associated with the position of the Altmühl and Swabian Rezat Rivers (Figs. 2 and 3), draining both parts of the European Watershed: the Altmühl River is a tributary of the Danube River that finally flows into the Black Sea. The modern mean water level of the Altmühl at its supposed confluence with the Fossa Carolina is 408.3 m a.s.l. [33]. Medieval navigability of the Altmühl close to the canal is documented in different Carolingian documents [33]. The Swabian Rezat River flows northward from Dettenheim to the east of the Fossa Carolina (Fig. 3) into the Main, a tributary of the Rhine that flows into the North Sea. During the Medieval period, the Swabian Rezat was at least navigable from Weissenburg downstream [33]. The Swabian Rezat was probably rerouted during the Carolingian period, turning it into an artificial channel. The present pond located in the Fossa Carolina is fed by a stream that originates from the western slopes of the Fossa Carolina, and is additionally filled up with seepage water. The outlet of the pond flows through Graben into the Altmühl.

## Methods

### LIDAR digital elevation model

High-resolution airborne laser scanning data (LIDAR) were supplied by the Bavarian land surveying office in Munich. These data were used to generate a digital elevation model (Fig. 3). We used the program ArcGIS 10 for data management and mapping.

### Flux gate magnetic survey

In the West-East Section of the Fossa Carolina a Bartington Grad601 fluxgate magnetometer was applied for a magnetic survey [41], [42]. Due to the difficult topography in the narrow zone between the two banks we used the handheld fluxgate magnetometer for the precise detection of the canal course. Grid measurement was carried out using a Topcon HiPer II DGPS device. Wooden canes mark the corners of each grid. PE-cords with metre spacing indicate northern and southern grid baselines. Additional PE-cords equipped with metre distance marks spanned between these two baselines were used as indicator for walking speed and position. For a higher resolution line spacing of 0.5 m and 4 measuring intervals per metre were selected resulting in a pixel size of 0.25×0.5 m. Since archaeological features with usually low magnetic anomalies are subject of this survey, the logged data range was limited to 100 nT, resulting in a sensitivity of 0.03 nT [42]. Georeferencing and patching of magnetic survey grids were handled in ArcGIS 10. For detailed analysis, processing and visualization of the data Geoplot 3.00 v software [43] was applied. The findings of the magnetic survey were used to determine locations for drilling.

### SQUID magnetic survey

To allow precise magnetic prospecting of large areas in the order of tens of hectares within reasonable time a motorized measurement system was developed [44]. The system based on SQUID (Superconducting Quantum Interference Device) sensors, which provide very high magnetic field resolution also during rapid traverses over the ground. The recorded magnetic maps have a centimetric resolution thanks to the SQUID sampling rates of 1000 measurement points per second, the used differential GPS technique (Trimble R8 GNSS) and data acquisition unit including very low noise and fast 24-bit A/D converters developed in-house. The detailed data post processing results in magnetic maps as well as a morphology map of high resolution. No data filtering has to be applied. The maps are *a priori* geo-referenced, which allows a precise and easy integration with aerial images and other GIS data.

The current setup of the system allows the synchronous recording of up to 12 SQUID gradiometer signals. Thus, several components of the gradient of the Earth's magnetic field up to the full tensor can be detected. This maximum of magnetic information is the basis of our approaches to calculate the possible subsoil distributions of the magnetic sources [45]. In this way, depth informations and three-dimensional shapes corresponding to the detected anomalies can be estimated in addition to the standard two-dimensional magnetic maps.

### Drilling campaigns

The flux gate and SQUID magnetic data were used to locate the canal course and to determine drilling positions. Our research group carried out drilling campaigns within the Fossa Carolina trench fills in 2012 and 2013 (Fig. 3), using an Atlas Copco Cobra Pro hammer and a 60 mm diameter open corer. Using this technique, we obtained 60 cores with lengths between 2 and 9 m. Drillings were levelled using a Topcon HiPer II DGPS device. The District Office of Weißenburg issued the permit for the drilling campaign. The study was partially carried out on private land. All owners were contacted personally by phone call, email or directly in the field to attain entry rights. All owners permitted access to their private lands and to take samples by the authors. The permission were recorded in an internal excel file after oral or written confirmation. Please contact the cities of Treuchtlingen and Weissenburg for future permissions and for the list of registered proprietors. The field studies (see Fig. 3 for GPS coordinates) did not involve endangered or protected species.

### Sedimentological analyses

We determined grain size distributions of each sediment layer to obtain information about the deposition process. This method is applicable to samples with a content of organic matter less than 5%. Bulk samples (10 g) were left in 50 ml 35-% hydrogen peroxide (H_2_O_2_) overnight, and heated to remove organic matter during the next day. Afterwards, the samples were dispersed using 10 ml 0.4 N sodium pyrophosphate solution (Na_4_P_2_O_7_) and ultrasonic treatment for 45 minutes. Grain size analysis of the sand fraction was carried out by means of the dry-sieving technique (2000–630 µm: coarse sand, 630–200 µm: medium sand, 200–125 µm: fine sand, 125–63 µm: finest sand). Coarse silt (63–20 µm), medium silt (20–6.3 µm), fine silt (6.3–2.0 µm), coarse clay (2.0–0.6 µm), medium clay (0.6–0.2 µm) and fine clay (<0.2 µm) were measured by X-ray granulometry (XRG) using a SediGraph III 5120 (Micromeritics) [46].

For calculating the content of organic matter, we measured the content of total carbon by using a CNS analyser vario EL cube (Elementar), and determined the content of inorganic carbon by calcimeter measurements (Scheibler method, Eijkelkamp). Resulting values of organic carbon were multiplied by 1.72 in order to obtain contents of organic matter [47].

### Numerical dating

Chronological information was obtained using ^14^C dating: Plant macrofossils (charcoal and wood) from clastic and peat layers were processed by accelerator mass spectrometry (AMS) (Tables 1 and 2). Radiocarbon ages were calibrated using quickcal2007 ver.1.5 [48].

### Classification of the organic-rich layers

Following Eckelmann [47], organic-rich layers were classified into peat and sapropel layers. The classification of organic-rich layers offers significant information about local hydrological conditions during phases of growth/sedimentation (e.g. fen facies, limnic facies). The degree of peat decomposition was determined following Von Post [47]. Degrees of decomposition depend on the preservation of plant residues, peat colour, colour of the water that is squeezed out and the condition of the remains after squeezing.

### Electrical resistivity tomography

Measurements of 2D and 3D electrical resistivity tomography were carried out in the West-East Section to detect boundaries of artificial ponds and potential Carolingian shipping chutes. Geophysical resistivity methods harness contrasts of the electrical conductivity σ or the electrical resistivity ρ (ρ = 1/σ) in sediments in order to detect different underground structures. Hence, buried archaeological features might be mapped under the precondition of existing resistivity contrasts in comparison to the surrounding sediment matrix. Generally, gravels and stones show increased resistivity whereas a clayey to silty texture is characterized by lower resistivity. To define the electrical characteristics of an underground volume metal probes (electrodes) are placed in the ground. Two probes are used in order to feed into the ground an electrical current. The resulting potential difference between two other probes is measured. By applying Ohm's law under consideration of the geometry of the electrode configuration the apparent electrical resistivity can be calculated. These values have to be transformed in a model of the spatial distribution of the resistivity by use of numeric inversion algorithms.

The electrical resistivity measurements were carried out using the multi-electrode system GeoTom MK1E100. Using this system up to 100 electrodes can be controlled. For data acquisition a pole-dipole configuration (”Half Wenner”) was used [49]. The spacing of the elctrodes was 1 m. The longitudinal profile was processed by means of the numeric inversion software DC2DInvRes [50].

## Results

### Types of sediment texture

**Figure 4:**
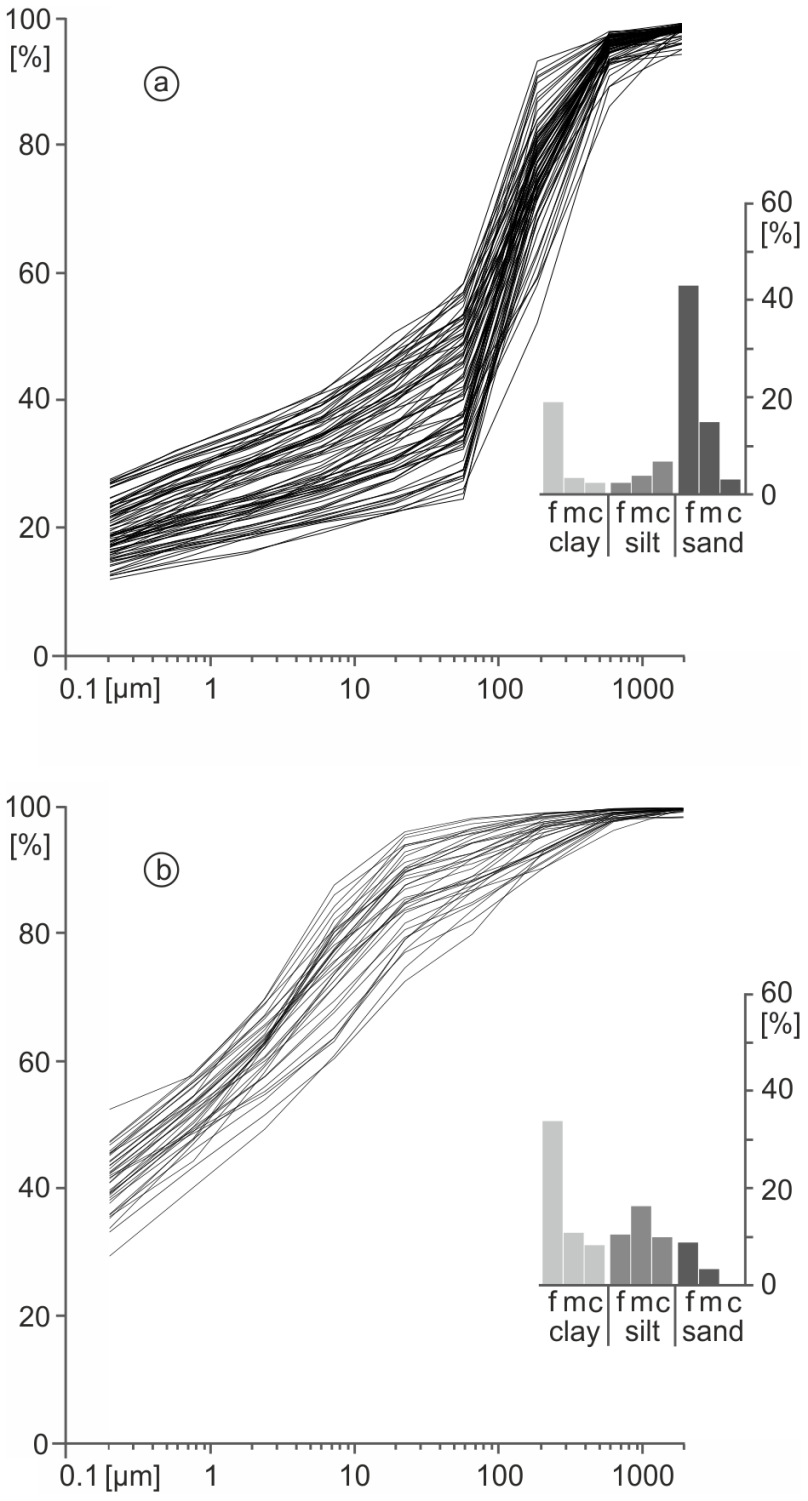
Types of sediment texture in the Central Section and the West-East Section. a) fluvial type and b) ponding type.

**Figure 5:**
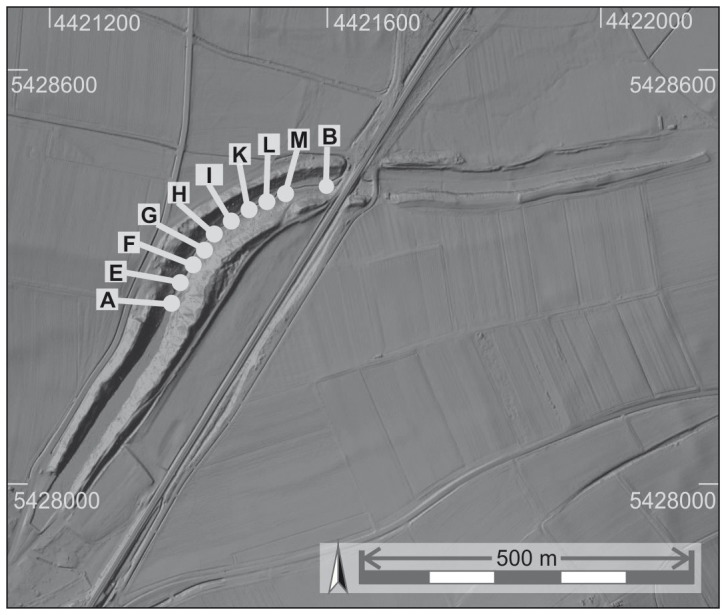
Digital elevation model of the Central Section including detailed drilling positions. Raw data source: LIDAR data are cordially provided by Bavarian Land Surveying Office for illustrative purposes.

Regarding the grain size data we were able to separate generally three types of sediment texture with characteristic grain size distributions. a) The ubiquitous ‘fluvial type’ sediment texture (Fig. 4a) reveals a bimodal distribution with peaks in medium to fine sand fraction as well as in the fine clay fraction. The majority of sediment samples from the Central Section and the West-East Section show this quite similar type of grain size distribution. We suggest that the trenches of the Central Section and of the West-East Section were excavated within this one fluvial grain size type only. The maximum in the medium to fine sand fraction can be explained by fluvial sediments which were transported by saltation and surface creeping [51]. Hence, the Carolingian and post-Carolingian ‘fluvial type’ trench fills represent re-deposited material from the banks which are build up with the originally Quaternary fluvial fills of the valley divide. b) The ‘ponding type’ sediment texture shows a bimodal distribution with peaks in medium silt and fine clay (Fig. 4b). For this second group we assume a deposition process typical for open water bodies, since the grain size distribution is characteristic for suspended load [51]. The ubiquitous sandy peak from the ‘fluvial type’ group is missing here. c) A third type of sediment texture, the ‘Miocene clay type’ indicates a strong maximum in the fine clay fraction. These thick and homogenous sediments were deposited during a long-term limnic phase. We detected this type of sediment texture only in drilling E (Fig. 5).

### Central Section

**Figure 6:**
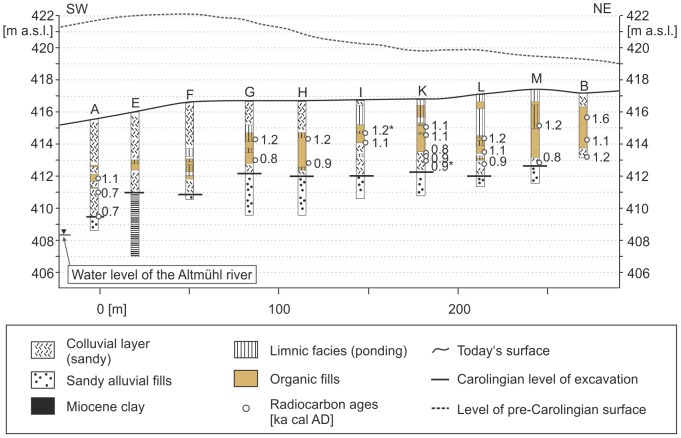
Chronostratigraphical documentation of the Central Section of the canal. Raw data from the cores A and B are published in Leitholdt et al. [17], those from the cores E, F, G, H and I are published in Leitholdt et al. [36]. The figure is not similar to formerly published figures.

On the basis of all core findings from the Central Section (Fig. 5), we are able to present the refill process of the Central Section in a synthetic chronostratigraphical form (Fig. 6). We can assume five principal phases of geomorphological or depositional relevance. ***I*** *)* The pre-Carolingian parent material in the Central Section consists of Miocene clays (drilling E only) and Quaternary fluvial fills of the valley divide. During the Quaternary the upper part of the valley floor was filled-up with well-stratified fluvial sediments poor in organic matter (File S1 and Fig. S2). These deposits feature generally a ‘fluvial type’ sediment texture (Fig. 4a). ***II*** *)* The 2^nd^ phase represents the excavation of the Carolingian trench. Our radiocarbon ages clearly indicate that the excavation started during the Early Medieval period. A *Quercus sp*. charcoal sample taken from a brownish layer at the base of core A shows an age of 691–759 cal AD (Table 1). The oxidized brownish layer in core A might feature the walking horizon during the time of Carolingian excavation. In core G and H we assume that the Carolingian excavation depth is below the level of two radiocarbon samples which yield ages of 793–879 and 827–939 cal AD (Fig. 6 and Table 1). Comparing all excavation depths in the Central Section, a stepwise altitudinal increase from core A up to core M is detectable (Fig. 6). In core A we have evidence for an abrupt refill of the excavated trench bottom. A *Quercus sp*. charcoal sample taken 1.5 m above the brownish layer indicates an age of 692–762 cal AD which is comparable with the result of a ^14^C dating from the brownish layer itself (691–759 cal AD, Table 1). This is quite similar to the observations in core K. Here, a probably re-deposited wood fragment features an age of 701–773 cal AD and covers ^14^C dated wood remains which reveal younger ages of 813–923 and 797–921 cal AD (Fig. S2 and Table 1). The Early Medieval age inversion at the lowermost sequence of core K (Fig. S2) suggests an abrupt refill process shortly after the excavation of the trench. ***III*** *)* The 3^rd^ phase indicates evidence for Carolingian ponds in the Central Section. In core H the sapropel layer between 412.28 and 412.35 m is located beneath a peat layer which reveals an age of 827–939 cal AD (Table 1). However, there is no evidence for Carolingian ponds in the majority of the cores of the Central Section. Indeed, at the trench bottoms of cores G and M we found Carolingian peat layers (793–879 cal AD and post 723–851 cal AD, Table 1) which show at least semi-terrestrial conditions but not clear evidence for Carolingian ponds. Numerous *Salix sp.* and *Alnus sp.* macro-remains indicate a local semi-terrestrial environment as well (Fig. S2). ***IV*** *)* The 4^th^ phase represents the main ponding and peat-growing phase within the Central Section. The deposits do not feature Carolingian but younger High Medieval ages between the 11^th^ and 13^th^ centuries AD (Table 1). Compared to the lower Carolingian deposits, the High Medieval sediments provide stronger evidence for ponds as indicated by sapropels and ponding-type sediment textures in almost all cores (E, F, G, H, I, K, L, M) (Fig. 6). ***V*** *)* The overlying terrestrial deposits above the High Medieval organic-rich layers characterize the final 5^th^phase in the Central Section which is probably still continuing today. In the uppermost sequence of the cores A, E, F, G and H (Fig. 6) the grain size distributions are similar to those found below the organic-rich layers which are dominated by the ‘fluvial type’ grain size group. Here, the sediments of the 5^th^phase originate from the Quaternary valley fills, feature re-deposited material from the adjacent Carolingian banks which were built up by Quaternary valley deposits. The uppermost clayey to silty sequence of cores I, K, L and M mirrors a suspension-load and partially organic-rich deposition process starting in the 13^th^ century AD (Fig. 6). We assume a High Medieval to modern ponding phases at this point.

### West-East Section

**Figure 7:**
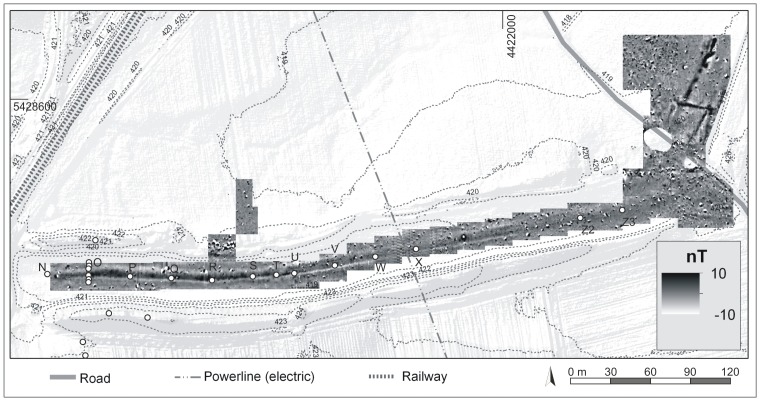
West-East Section of the Fossa Carolina. The figure shows a digital elevation model with detailed drilling positions, electrical transects, and the flux gate magnetic survey. White dots mark the drilling positions. The black lines show the geo electrical transects 1 and 2. Raw data source: LIDAR data are cordially provided by Bavarian Land Surveying Office for illustrative purposes.

**Figure 8:**
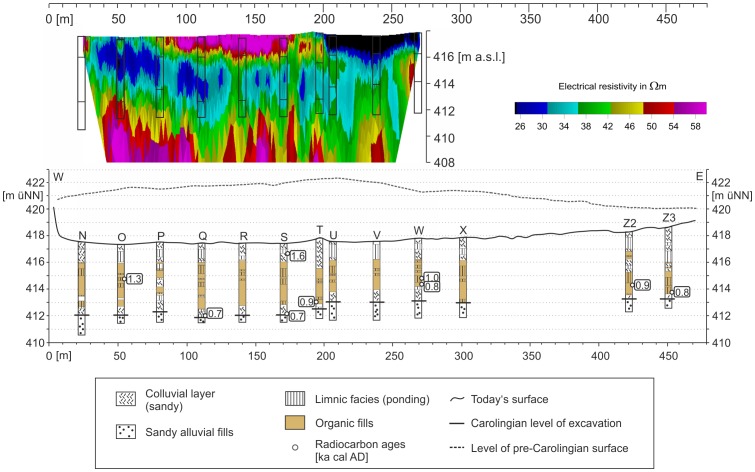
Longitudinal profile of the West-East Section including an electrical resistivity profile.

The magnetogram of the West-East Section reveals a slight positive, elongate anomaly which is parallel to the direction of the banks but 2.5 m north of a current drainage trench. The anomaly is particularly noticeable in the western part of the West-East Section (Fig. 7) and became gradually more diffuse in the eastern part. In the western part the total width of the anomaly is between 6 to 10 m. Due to the elongate and narrow form of the anomaly we assumed here the course of the Carolingian canal. As the course of the canal should be constrained by *in situ* findings and ^14^C dating, cores were drilled exactly in the centre of the anomaly with consistent intervals of 30 m. Of specific interest were an interruption and a slightly displacement of the anomaly between the cores S and U. At this point we assumed a subdivision of the canal, probably due to a favoured cross bank or shipping chute. Putting all core findings from the West-East Section together, we document the deposition processes of the West-East Section in a synthetic chronostratigraphical context (Fig. 8). Comparable to the Central Section we postulate five principal phases of geomorphological or depositional relevance for the West-East Section: ***I*** *)* the drilled pre-Carolingian parent material in the West-East Section consists of Quaternary fluvial fills of the valley divide. The stratified fluvial sediments are poor in organic matter and reveal a bimodal grain size distribution with a typical maximum in medium to fine sand and a secondary peak in fine clay. ***II*** *)* The 2^nd^ phase reflects the excavation of the Carolingian trench. The trench bottom in the West-East Section is generally characterized by the distinct onset of plant macro-remains (Fig. S3) indicating earliest trench refill processes. Three wood remnants in cores T, W and Z3 were found slightly above the detected trench bottoms, and all ^14^C ages are in the range of the Carolingian period (819–933, 792–870 and 716–836 cal AD, Table 2). Two wood remnants from the trench bottom at cores Q and S reveal calibrated ^14^C ages of 689–761 and 666–708 cal AD. Both ages are 70 to 100 years older than the historical documented onset of canal construction in 793 AD [24]. Two pieces of *Quercus sp.* suggest the use of timber for stabilizing the excavated trench. Comparing all excavation depths in the West-East Section, cores N to T show levels of 412.24 m with very small deviations of only ±0.31 m (Fig. 8). We interpret the small deviations as evidence for a planned construction of a pond at this site. The cores U to Z3 mirrors more shallow excavation depths between 413.00 and 413.30 m (Fig. 8). Hence, in the West-East Section there is evidence for one step-like increase of the trench bottom between cores T and U. Regarding the findings from the electrical survey, there is no evidence for a cross bank or shipping chute between the detected trench bottoms in core S and U (Fig. 8). ***III*** *)* The 3^rd^ phase indicates evidence for Carolingian ponds in the West-East Section. On the one hand the presence of ‘fluvial type’ clastic deposits, with visible plant macro-remains at the base of the trench fills in cores N, O, P, Q, and W, points to an initial colluvial re-deposition process shortly after the time of excavation. On the other hand we have strong evidence for ponds in most of the lowermost trench fills of the West-East Section. Here, cores Q, T, W, X, Z2 and Z3 reveal ‘pond type’ sediment texture or sapropel layers at the canal bottom. The presence of alternating peat and sapropel layers and the abundance of *Salix sp.* and *Alnus sp.* macro-remains points to open water bodies with adjacent semi-terrestrial zones of fen and swamp forest during the Carolingian period. According to the ^14^C data from the lower trench fills, we have evidence for this type of canal ecosystem from the Carolingian period to at least 990–1020 cal AD (core W, Table 2). The electrical survey features a continuous layer of low electrical resistivity between the cores N and V (green and blue colours, Fig. 8). In contrast to the different trench bottom levels in the western and eastern part of the West-East Section, there is no evidence for isolated ponds from the magnetic survey. However, a wooden Carolingian weir would not be necessarily visible in the electrical resistivity profile. ***IV*** *)* The 4^th^ phase represents an enduring ponding and peat-growing phase within the West-East Section. Compared to the Carolingian deposits, the High Medieval sediments reveal even more evidence for ponds as indicated by sapropels and ponding-type sediment textures in all cores (N, O, P, Q, R, S, T, U, V, W, X, Z2 and Z3) (Fig. 8). Chronologically, we have evidence for this ponding-rich environment up to the 13^th^ century AD (core O, Fig. 8, Table 2). ***V*** *)* The clastic sediments above the medieval peat and sapropel layers represent the final 5^th^ phase in the West-East Section which is probably running until today. In the western part of the West-East Section the grain size distributions are equivalent to those found below the organic-rich layers which are dominated by the ‘fluvial type’ sediment texture. Here, the medium to fine sandy sediments of the 5^th^ phase originate from the Quaternary valley fills, respectively feature re-deposited material from the adjacent Carolingian banks which were built up by Quaternary valley deposits. The sandy texture is also indicated by a high electrical resistivity (Fig. 8). In the eastern part of the West-East Section a final clayey to silty sequence of cores U, V, W, X, and Z2 indicates a suspension-load deposition process. Although the matrix of the final sequence is reduced in organic matter, we assume evidence for a former open water body due to the analysed ‘ponding type’ sediment texture which was identified in all sediment samples. This compact facies is also visible in the low electrical resistivity values (Fig. 8).

**Figure 9:**
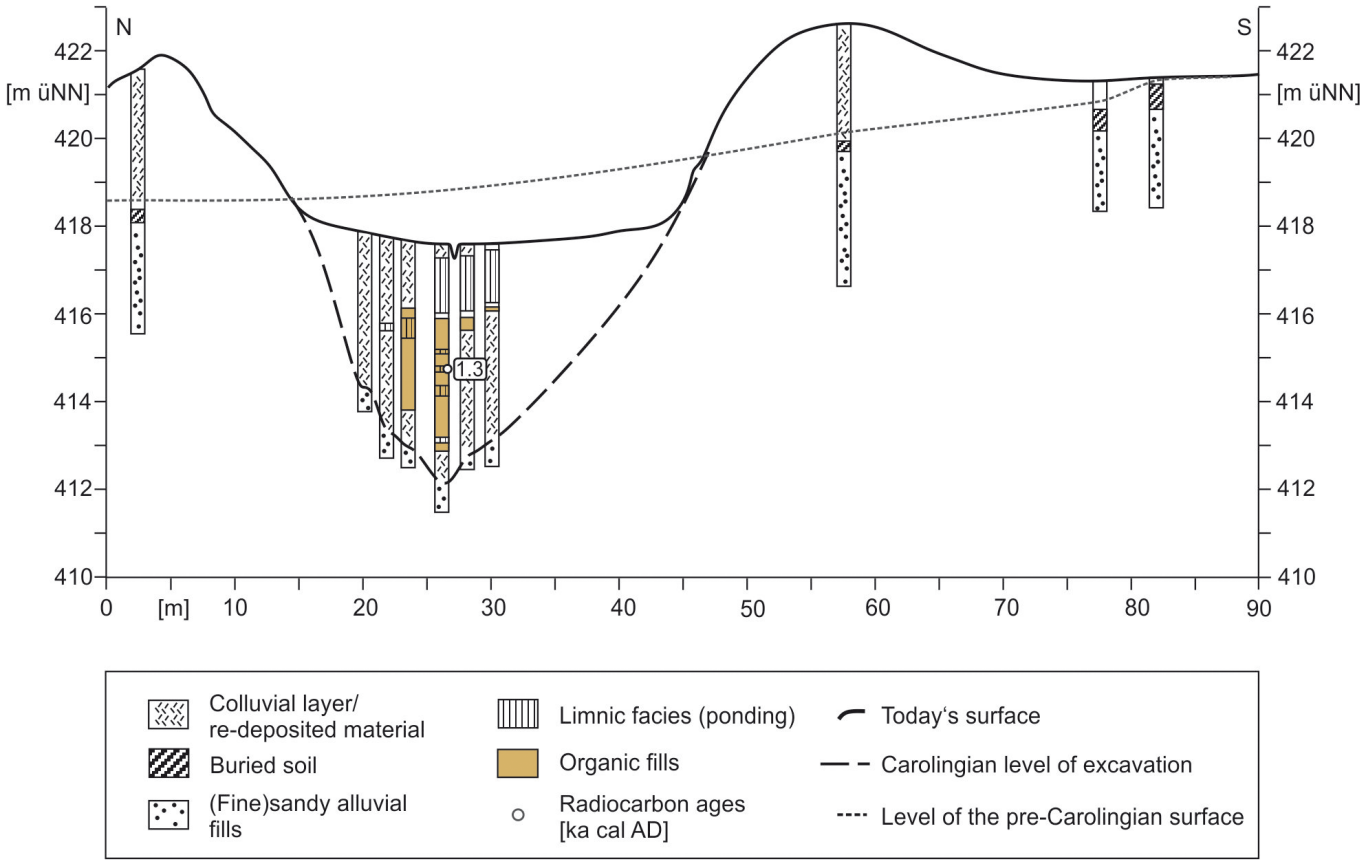
Cross section of the West-East Section at core O position.

Fig. 9 shows a cross section of the West-East Section at core O site. The drilling positions are plotted in Fig. 7. The chronostratigraphical findings reveal evidence for a planned canal width of around 5 to 6 m. Only in this narrow zone sapropel layers and organic fills are detectable. Surprisingly, we have evidence for a narrow zone of ponding and semi-terrestrial conditions not only for the Early Medieval strata but also for all younger sequences in the cross section (Fig. 9).

### Northern Section

**Figure 10:**
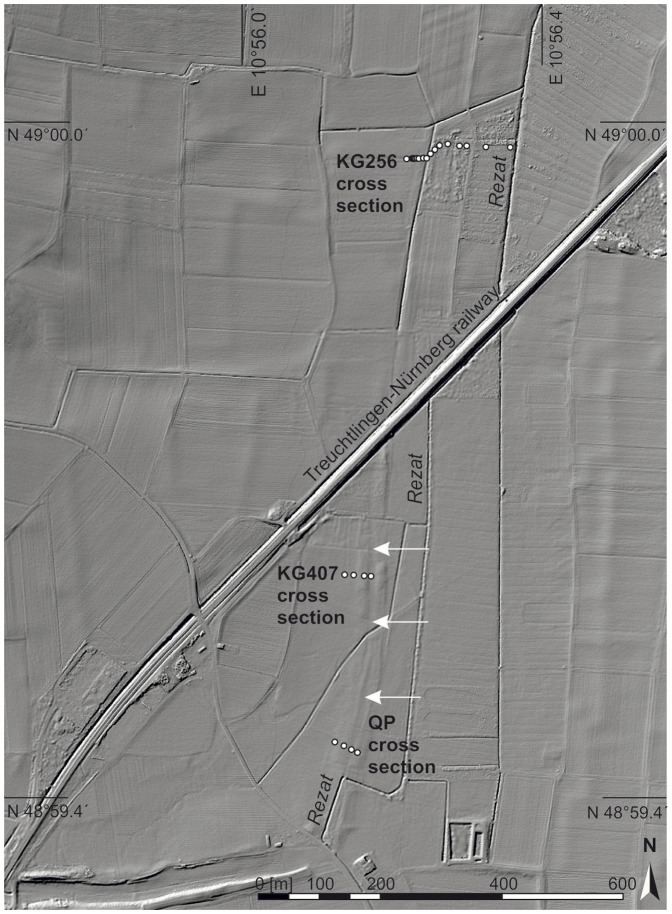
Airborne LIDAR scan with drilling positions in the Northern and North-Eastern Sections. White arrows indicate the assumed course of the canal. The cross sections are documented in Figs. 14, 15, and 16. Raw data source: LIDAR data are cordially provided by Bavarian Land Surveying Office for illustrative purposes.

**Figure 11:**
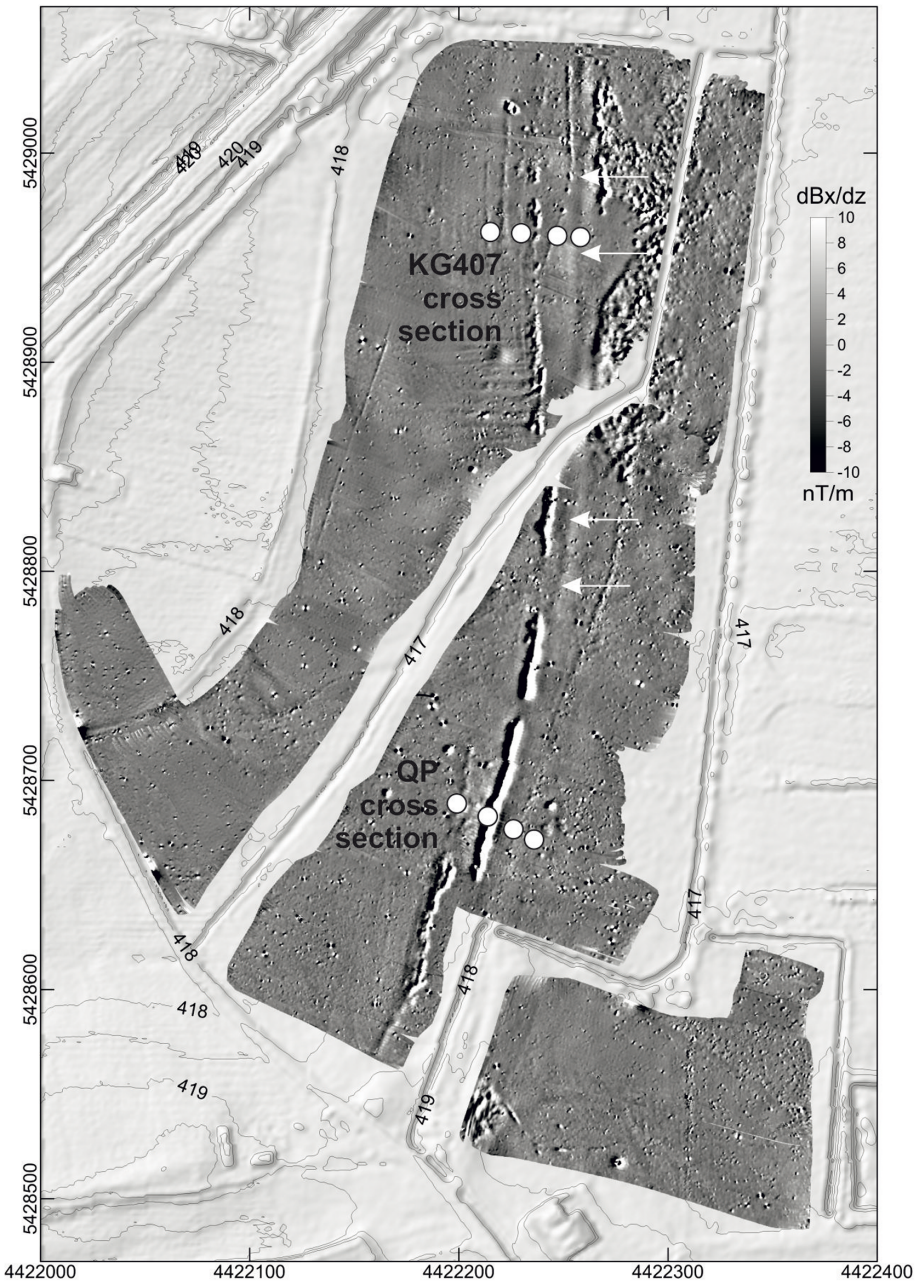
SQUID magnetogram from the Northern Section. White dots mark the drilling positions. White arrows feature the course of canal. The image represents an area of 600 m×400 m. The magnetogram is embedded in a LIDAR illustration, which raw data are cordially provided by Bavarian Land Surveying Office.

The Northern Section covers the probable course of the canal from the present-day connection road between Grönhart and Dettenheim to the second crossing of the Treuchtlingen-Nuremberg railway (Fig. 3). Hence, there must be a quite angular bend of the canal course in the transition from the West-East Section to the Northern Section (Fig. 3). The LIDAR digital elevation model from the Northern Section features two banks orientated in North-South direction (Fig. 10). The bank remnants are hardly visible in the field but the digital elevation model clearly documents evidence for an anthropogenic structure. The distance between the two bank crests varies only little between 25 and 30 metres (Fig. 10). Hence, this structure might reflect the extension of the Fossa Carolina in northern direction as already assumed by Röder [52] and later on by Koch and Leininger [33]. The SQUID geomagnetic data strongly support this assumption. Pronounced linear magnetic anomalies with nearly North-South direction are detectable within the complete North-South extension of the Northern Section (arrows in Fig. 11). The linear anomalies with the strongest signal of more than 80 nT/m (sharp black-white contrast in Fig. 11) and a total length of about 250 m occur between a sharp 90 degree turn of the current Rezat course in the south and a modern drainage trench in the north. However, these anomalies do not mark the principal canal course but they seem to flank the west side at a distance of 10 to 20 m. First calculations of the corresponding subsoil structure from the magnetic data show a narrow shape with a depth of about 1.5 m, which is in good agreement with the below presented drilling findings. Based on the detected magnetic anomalies and the LIDAR data we have chosen drilling sites for proving the course of the canal in the Northern Section. Two cross sections (Fig. 10 and 11) were selected for the stratigraphical documentation of the alluvial valley deposits, the trench bottoms, the banks and the trench fills. For the first time we sampled wood remnants at the trench bottom to prove the Carolingian age of the canal in the Northern Section.

**Figure 12:**
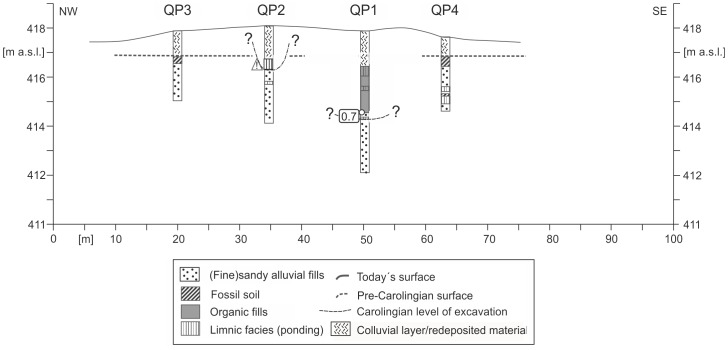
Stratigraphical documentation of the QP cross section within the Northern Section of the canal. The drawing shows a 42 m long cross section with the different facies types and the Carolingian level of excavation.

QP cross section (Fig. 12) consists of four cores (QP1 to QP4). QP1 is exactly positioned in the assumed canal course. A charcoal sample (*Quercus sp.*) from the trench bottom features a calibrated ^14^C age of 656–670 AD (Table 2). This age is around 130 years older than the historically documented canal construction at 793 AD [24]. However, keeping possibly matured oak wood in mind, the ^14^C sample supports the hypothesis of the Carolingian age of the trench bottom and indicates evidence for the use of timber in an advanced Carolingian construction phase. Above the Carolingian trench bottom the QP1 core features a 220 cm long sequence of predominantly organic trench fills. The fills consist of peat and sapropel layers indicating local semi-terrestrial and limnic deposit conditions. The upper part of QP1 core is characterized by a covering organic-poor colluvial layer of 120 cm thickness. The lower part of QP3 and QP4 cores reveals sandy alluvial fills of the Rezat valley predominantly poor in organic matter. The sandy fills are covered by an *in situ* half-bog A horizon featuring the Carolingian surface and the edge of the close by Rezat fen. The A horizon is buried by 60 to 100 cm of excavated trench material. QP2 core is exactly positioned in the strong magnetic anomaly (Fig. 11). The lower part of the QP2 sequence represents organic-poor sandy Rezat alluvial fills. The alluvial fills are covered by a black and a red (2.5 R) clayey to silty layer (130 to 170 cm below surface). These layers reveal extremely high values of volume specific magnetic susceptibilities (40,000 to 53,000 10^−6^ SI units). The formation of these layers remains unclear so far. As the black and red clayey to silty layers and the capping reddish-brownish colluvial layer (0 to 130 cm) seem to be incised in the half-bog A horizon as well as in the bank remnants we assume a Carolingian to post-Carolingian age of the magnetic anomaly.

**Figure 13:**
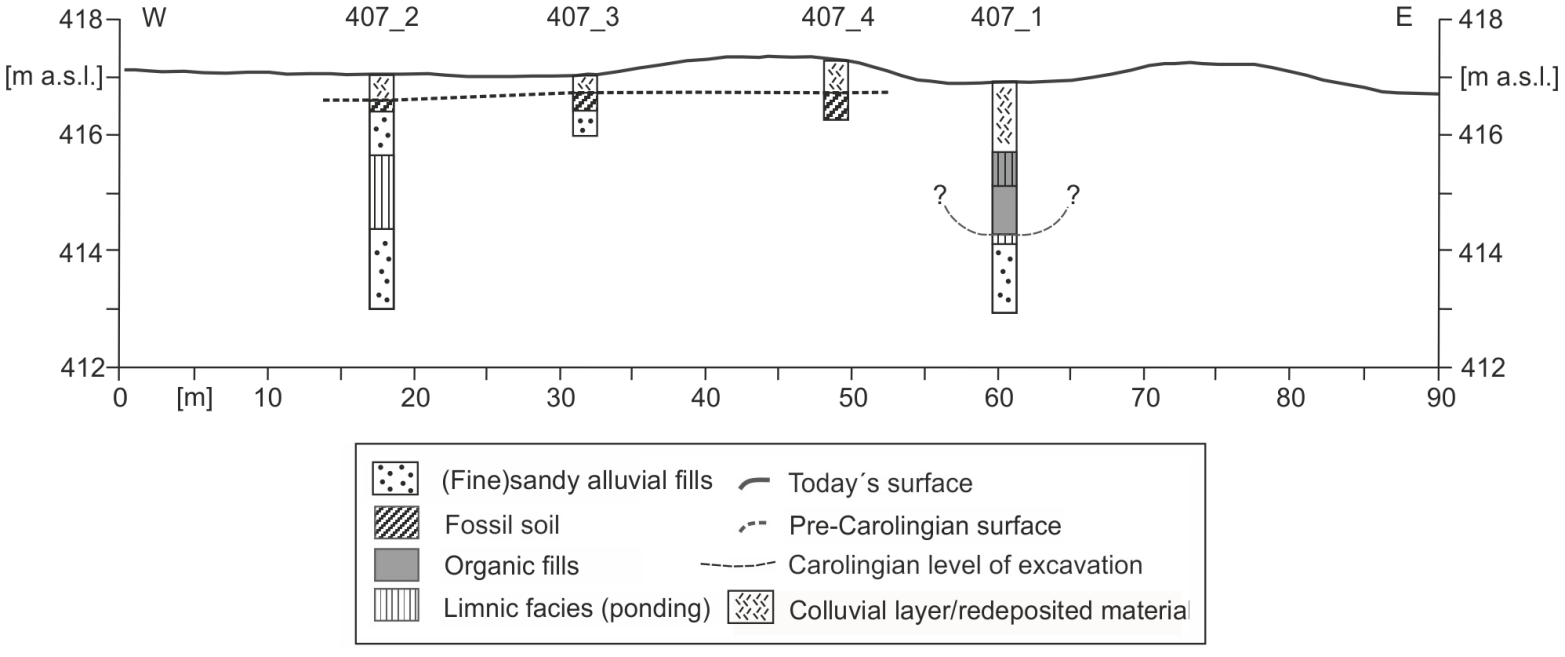
Stratigraphical documentation of the KG 407 cross section within the Northern Section of the canal. The drawing shows a 90 m long cross section with the different facies types and the Carolingian level of excavation.

KG-407 cross section (Fig. 10 and 13) consists of four cores (KG-407-1 to KG-407-4). KG 407-1 is exactly positioned in the assumed canal course. The lower sequence reveals sandy fluvial fills (Fig. 13). The greyish layer is poor in organic matter and fines up to a covering greyish clay layer. The middle sequence is characterized by peat and sapropel layers which are interpreted as fills of the Carolingian trench. The layers indicate a semi-terrestrial to limnic environment. A ^14^C sample from a seed of the peat layer (137 cm below surface) features a calibrated age of 1444–1464 AD (Table 2). Hence, the canal exhibited an open body of water for at least 650 years, assuming a Carolingian age of the excavated trench at this point. KG 407-2 core represents a sequence of the undisturbed fluvial fills. The lower part of the sequence features organic-poor fine-sandy deposits. The medium sequence shows clayey layers with shallow organic-rich interruptions. Similar to the QP cross section the fluvial sequence ends with an *in situ* half-bog A horizon due to the adjacent Rezat fen. The half-bog A horizon is buried by a sandy bank featuring the former excavated trench material. The half-bog A horizon and the covering bank material can also be detected in the cores KG 407-3 and KG 407-4 (Fig. 13).

### North-Eastern Section

**Figure 14:**
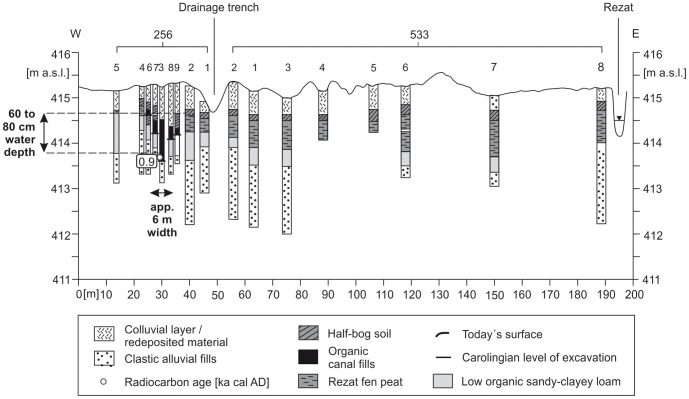
Stratigraphical documentation of the KG 256 cross section within the North-Eastern Section. The cross section shows the in situ layers of the Rezat fen in the East and the trench structure in the West. For the first time there is in situ evidence for a Carolingian trench in the North-Eastern Section. The trench bottom is shown by horizontal bars.

Within the North-Eastern Section the course of the canal is unknown. Whereas in the south of the Nuremberg-Treuchtlingen railway the topography slightly mirrors the remnants of two parallel banks (Fig. 3), these banks are not any more visible in the North-Eastern Section. Additionally, no *in situ* dating from a possible trench bottom is available yet which might prove the presence of a Carolingian canal here. However, linear North-South straightened magnetic anomalies are also detectable in the North-Eastern Section close to the railway where no topographic features are detectable by LIDAR. Thus, we conclude that the assumed Carolingian canal course is revealed and precisely marked by these magnetic signatures. Fig. 10 shows the drilling positions of our examined KG 256 cross section through the Rezat fen in the North-Eastern Section. At the bottom of the cross section medium to fine-sandy greyish fluvial deposits fines up to a greyish sandy loam poor in organic matter. The sandy loam is covered by a 50 to 100 cm thick organic layer featuring slightly decomposed Rezat fen peat deposits. The peat layer terminates with a half-bog *in situ* A horizon, which indicates high decomposition of the upper peat layer. Between a modern drainage trench and the present-day straightened course of the Rezat River in the East (Fig. 14) the half-bog A horizon is covered by a colluvial-like brownish sandy loam.

In the west of the modern drainage trench the slightly decomposed peat layer is absent. In contrast, the organic-rich material in the cores 256–3, –6, –7, –8 and –9 (Fig. 14) reveals no clear structures of botanical remnants. In core 256–3 the organic-rich layer attains an extraordinary thickness and depth, which does not match with adjacent cores 256–7 and 256–8. Furthermore, the ubiquitous basal organic-poor sandy loamy layer is absent here. In conclusion, we postulate purely from the stratigraphical findings an excavated basal sandy loamy layer which was subsequently filled up with organic-rich trench deposits. Thus, we are sure that we detected an artificial sedimentological sequence here which might correspond to a former canal construction. We have clear evidence for a Carolingian trench based on the result of a radiocarbon dating. A wood macro-remnant from the trench bottom reveals a calibrated ^14^C age of 829–927 AD.

## Discussion

### Evidence for Early and High Medieval ponding

Grain-size analyses, the classification of the organic layers, and the chronostratigraphical documentation of the trench fills of the Central Section and of the West-East Section clearly show that the fills can be divided into separate depositional units. The banks and the colluvial deposits at the base of the trench are made of the same material that constitutes the underlying basal Quaternary fluvial deposits of the region. In contrast, there is strong evidence for clayey to silty layers in the trench fills which reveal remnants of suspension limited stillwater deposition and, therefore, the evidence of Carolingian and post-Carolingian ponding (Table 3). These findings are strongly supported by numerous sapropel layers in the Central Section as well as in the West-East and Northern Sections. Hence, there is no doubt about the existence of ponds at the Fossa Carolina during the Early and High Medieval periods (Table 3). Chronological data of previous Fossa Carolina archaeological studies were limited to historical records and not supported by radiocarbon dating. Thus, in previous surveys all organic-rich layers were classified as a signal for a Carolingian canal [33]. For the first time, our data indicate enduring ponding phases from the Carolingian period up to the High Medieval period. We have evidence that at least isolated zones of the Fossa Carolina exhibit open water bodies for hundreds of years. We have to consider the enduring use of the ponds for fisheries as well.

**Table 3:** Geoarchaeological findings of the different canal sections: summarizing overview.

Canal section	Early Medieval trench	Early Medieval ponds	High Medieval ponds	Timber use	Chute	Weir	Abrupt Carol. refills	Stepped ponds	Summit canal concept	Number of drillings
(I) Central	+	+	+	+	o	o	+	+	+	10
(II) West-East	+	+	+	+	-	(+)	(+)	+	+	23
(III) Northern	+	+	+	+	o	o	o	(+)	+	8
(IV) North-Eastern	+	(+)	(+)	o	o	o	o	(+)	+	17
(V) Rezat Headwater [31]	o	-	-	o	o	o	o	o	(+)	2
(VI) Altmühl floodplain	o	o	o	o	o	o	o	o	o	Ongoing study

A plus sign (+) indicates clear *in situ* stratigraphical and chronological evidence, a circle (o) feature additional research needs, and a minus sign (-) indicates no *in situ* evidence.

Regarding the ^14^C data from the Central Section and the West-East Section, major peat growing phases and periods of sapropel accumulation were predominant during the 9^th^ to the 13^th^ centuries. Here, the Early Medieval ponding took place mainly during the 9^th^ century, and the High Medieval sapropels are dated between the 11^th^ and 13^th^ centuries.

### Evidence for abrupt refill processes and timber use

Numerous basal trench fills from the Central Section point to an abrupt refill process, starting immediately after the Carolingian excavation phases. We have evidence for at least 1 to 1.5 metres of Carolingian re-deposited material in cores A and K. Especially in core K the Early Medieval age inversion (Fig. 6 and Table 1) at the lowermost sequence points to an abrupt refill process shortly after the excavation of the trench. These findings correspond with the historical sources which describe a collapse of the Carolingian canal project due to unconsolidated bank materials slipping back in the trench after heavy rainfalls [24]. However, the reconstructed Early Medieval open water bodies in numerous core stratigraphies do not support a definite collapse theory.

It is striking that the analyses of the wood macro-remains indicate numerous *Quercus sp.* at the bottom of the trench fills (e.g. cores A, Q, S, and QP1). All ^14^C ages of these *Quercus* samples reveal ages slightly before 793 AD (Tables 1 and 2). We conclude that the dated *Quercus* samples from the trench bottom reveal evidence for timber use and mirror the advanced construction level of the canal. This corresponds with archaeological findings from the Northern Section. Here, a recent archaeological excavation suggests the use of *Quercus* boards for the stabilisation of the Carolingian canal edges [53].

### Early Medieval inland navigation requirements

Two geoarchaeological cross sections from the West-East Section and from the North-Eastern Section set limits for the required width of the planned Carolingian canal. Both cross sections reveal evidence for a conceptual width of the open water body between 5 to 6 metres. In the North-Eastern Section the maximum stage of the Carolingian water body can be limited by the level of the adjacent half-bog A horizon which features at least seasonal terrestrial conditions. Keeping this level in mind, the maximum water depth of the canal in the North-Eastern Section is limited to 60 to 80 cm. A width of 5 to 6 metres and a depth of 60 to 80 cm allow a crossing way passage of boats with a maximum width of about 2.50 m and a maximum draft of about 50 cm which corresponds to excavated Carolingian cargo scows with a payload of several tons [13], [54]–[57]. Although the final development of the entire waterway between the Altmühl and Rezat rivers is not proven yet, the reconstructed canal widths provide helpful information for the improvement of knowledge about Early Medieval inland navigation requirements in Central Europe. So far, there has been only few data available about the navigability of small inland waterways in Central Europe [10], [19], [58]. A couple of years ago, an Early Medieval hythe was discovered and excavated at the adjacent southern Franconian Schwarzach River, a small tributary of the Altmühl River [11], [59]. The headwater of the Schwarzach River is also limited by a valley divide of the Central European Watershed and hydrologically comparable with the Rezat River. Hence, there is evidence for the general integration of the small Franconian rivers and streams in the regional inland shipping networks across the Central European Watershed.

### Summit canal: advanced construction level of the known Carolingian canal course

**Figure 15:**
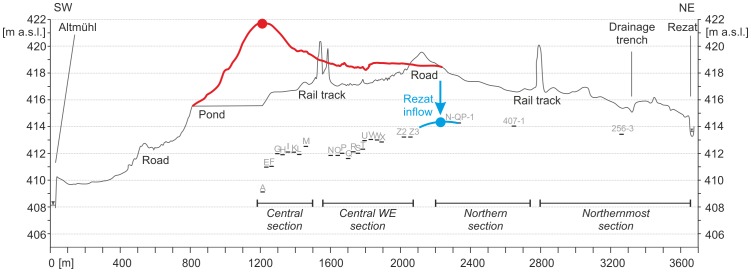
Synthetic longitudinal profile of the Fossa Carolina from Graben to Rezat River. a) The red line shows the Pre-Carolingian surface and watershed, the red dot indicates the summit the natural watershed. b) The short horizontal bars feature the Carolingian levels of excavation deduced from the drilling stratigraphies, and the blue dot indicates the artificial summit of the Carolingian trench bottom. c) The solid black line represents the level of the recent trench surface following the course of the canal.

On the basis of our drilling findings we are able to reconstruct a Fossa Carolina longitudinal profile with the levels of Carolingian excavation depths from Graben village to the Rezat River (Fig. 15). It is considerable that the excavation depths decrease along the Central and West-East course of the canal. In the zone of the Central European Watershed (reddish line in Fig. 15) the level of the trench bottom does not follow the level of the natural watershed. Here, the canal features maximum Carolingian excavation depths which correspond to maximum size of the adjacent banks. This clearly shows that a summit of the canal was not planned in the zone of the natural valley watershed. This required an additional Carolingian work load of enormous extent and we need a hydro-engineering explanation here. When we prolong the longitudinal profile into the Northern and North-Eastern Section, we have evidence for a summit canal concept and that the summit of the Carolingian canal is located around 1000 metres further to the East compared with the position of the natural watershed (Fig. 15). Here, we postulate a planned hydro-engineering concept. The artificial displacement of the watershed allows the opportunity to supply the summit of the canal with water from the Rezat headwater (Fig. 3 and Fig. 15). The Rezat spring reveals strong seasonal amplitudes in discharge due to the weak water yield of the shallow karst aquifers in this zone of the Franconian Jura [60], [61]. However, considering recent hydro-climatic conditions [62], [63] and summer discharge gauging the Rezat spring features a base flow of 6 l/s in the zone of the summit of the canal. This corresponds with a minimum daily water supply of around 500 cubic meters.

Although recent geoarchaeological research discards Koch's [15] reservoir theory for the Rezat headwater [35] the discovery of the trench bases clearly shows how the Carolingian breaching of the watershed took place and provides new evidence for the outstanding Early Medieval knowledge in hydro-engineering. Apart from the Fossa Carolina summit canal, there is only poor evidence for shipping canals in Early Medieval Europe. The Early Medieval Kanhave canal on Samsø Island is an exception [64]. However, historical sources generally document Early Medieval knowledge in the construction of mill canals and aqueducts in Central Europe [25], [65]–[68]. Even in the adjacent Paar floodplain and in tributaries of the Altmühl River we have archaeological evidence for mill canal construction and Early Medieval competence in hydro-engineering [11], [69].

### European dimension of the project: extension of the canal course to the north

Due to its extraordinary geomorphological favourableness the valley watershed between the Swabian Rezat and Altmühl Rivers played an important role in the Early Medieval trade and transport system. Here, the comparatively easy crossing over the European Watershed is characterised by a channelisation of supra-regional transport axes [29]. During the Carolingian era the Main-Rednitz-Regnitz-Swabian Rezat headwater system was equipped with a chain of royal courts and monasteries reflecting a central role for supra-regional trading and transport [16], [70]. Initiating with the Carolingian royal court at Hallstatt the succeeding courts upstream at Forchheim, Fürth, Roth and Weissenburg represent daily stages of an inland shipping or trading route towards the European watershed [19]. The supra-regional importance of crossing the land passage between the Swabian Rezat River and the Altmühl River justifies the enormous effort put in the construction of a Carolingian canal [1], [25], [71]. The geoarchaeological results from the Central and West-East Sections indicate an extraordinarily advanced Carolingian construction level of the canal (Table 3). Additional drilling campaigns in the Altmühl floodplain and archaeological excavations are required to clarify the location of the connection between the canal and the Altmühl River. However, our new SQUID geomagnetic data and geoarchaeological *in situ* findings from the Northern and North-Eastern Section prove for the first time the extension of the canal course in a northerly direction close to the confluence with the Rezat River. This leads to a total length of the constructed Carolingian canal of at least 2300 metres.

## Conclusions

The trench fills of the Fossa Carolina indicate numerous ponding phases during the Early and High Medieval periods (Table 3). We have evidence that at least isolated zones of the Fossa Carolina exhibit an open body of water for hundreds of years. The basal sequences of the trench fills feature occasionally sandy layers rich in isolated plant and wood remnants. These layers feature ^14^C age inversions and might be the result of abrupt and unintentional refilling processes shortly after the Carolingian construction phases. However, these abrupt refills are often covered by sediment sequences which indicate the later evidence for open water bodies. Hence, there is no clear evidence for an abrupt and final collapse of the Carolingian canal construction.

The reconstruction of two canal cross sections indicates a width of approximately 5 to 6 metres and a depth of 60 to 80 cm for the open water bodies. This would have allowed a crossing way passage in the West-East and Northern Section of the canal for Carolingian cargo scows with a payload of several tons. The synthesized longitudinal profile of the trench fills indicate clear evidence for a summit canal as the final hydro-engineering concept. We conclude that the artificial and labour intensive displacement of the watershed allows the opportunity to supply the summit of the canal with Rezat spring water. We detected an extraordinarily advanced construction level for a navigable waterway which mirrors the enormous Carolingian effort in crossing the Central European Watershed and in improving supra-regional inland navigation networks.

## Supporting Information

Figure S1 **Geological sketch of the study area.** Redrawn [39], [74].
(TIF)

Figure S2 **Chronostratigraphical documentation of core K.** Anthracological findings from the Central Section are presented in a synthetic form and normalized to the levels of core K. The black arrow marks the level of the Carolingian excavation depth.
(TIF)

Figure S3 **Stratigraphical documentation of core O.** Anthracological findings from the West-East Section are presented in a synthetic form and normalized to the levels of core O. The black arrow marks the level of the Carolingian excavation depth.
(TIF)

Table S1 **Technical and historical terminology related medieval inland navigation and canalisation.** The table shows specific terms which are used in the manuscript in a stringent and standardised form. The High Medieval Period from the late 10^th^ century to early 14^th^ century corresponds to the Central Middle Ages following the Oxford terminology [75].
(DOCX)

File S1 **Core K and O: examples of our detailed chronostratigraphical approach.** We show two representative cores from the Central Section and the West-East Section to document our stratigraphical approach in a more detailed form.
(DOCX)
