# Supplementary figures and images for: Charlemagne's Summit Canal: An Early Medieval Hydro-Engineering Project for Passing the Central European Watershed
Source: PLoS One. 2014 Sep 24;9(9):e108194. doi: 10.1371/journal.pone.0108194 (PMC4177111; doi:10.1371/journal.pone.0108194)

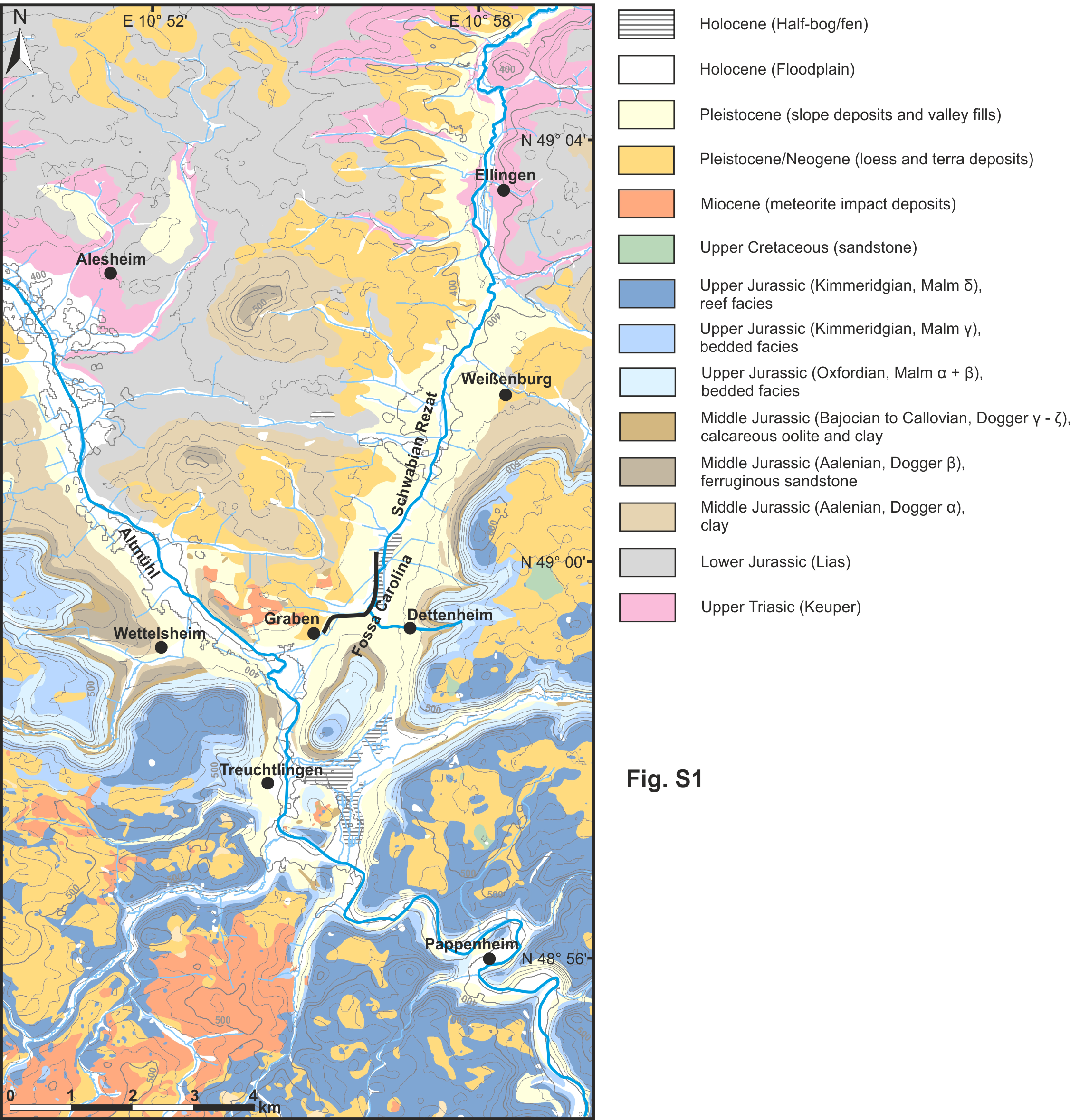

Supplement: Figure S1 — Geological sketch of the study area. Redrawn [39], [74]. (TIF) [file pone.0108194.s001.tif]

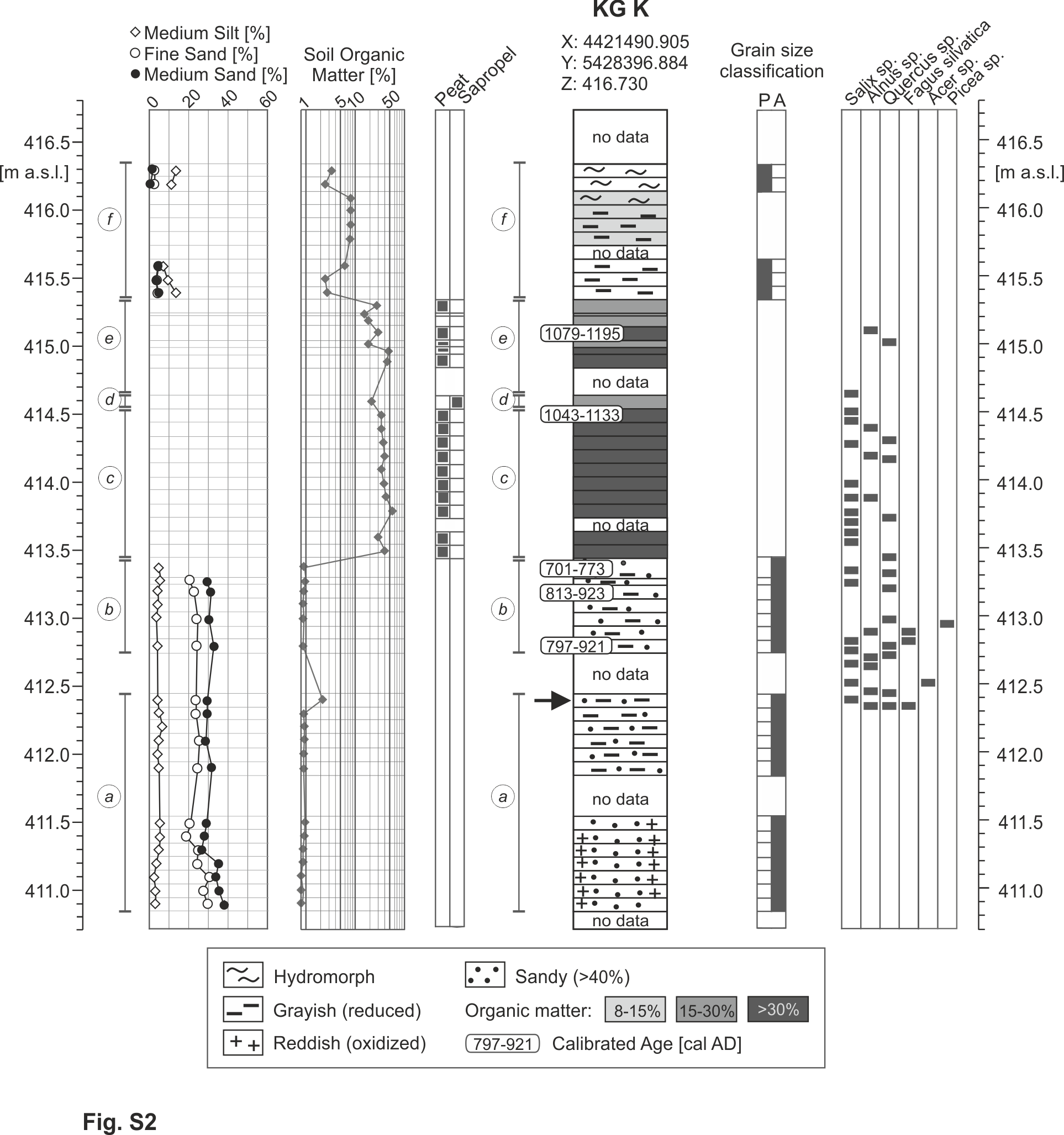

Supplement: Figure S2 — Chronostratigraphical documentation of core K. Anthracological findings from the Central Section are presented in a synthetic form and normalized to the levels of core K. The black arrow marks the level of the Carolingian excavation depth. (TIF) [file pone.0108194.s002.tif]

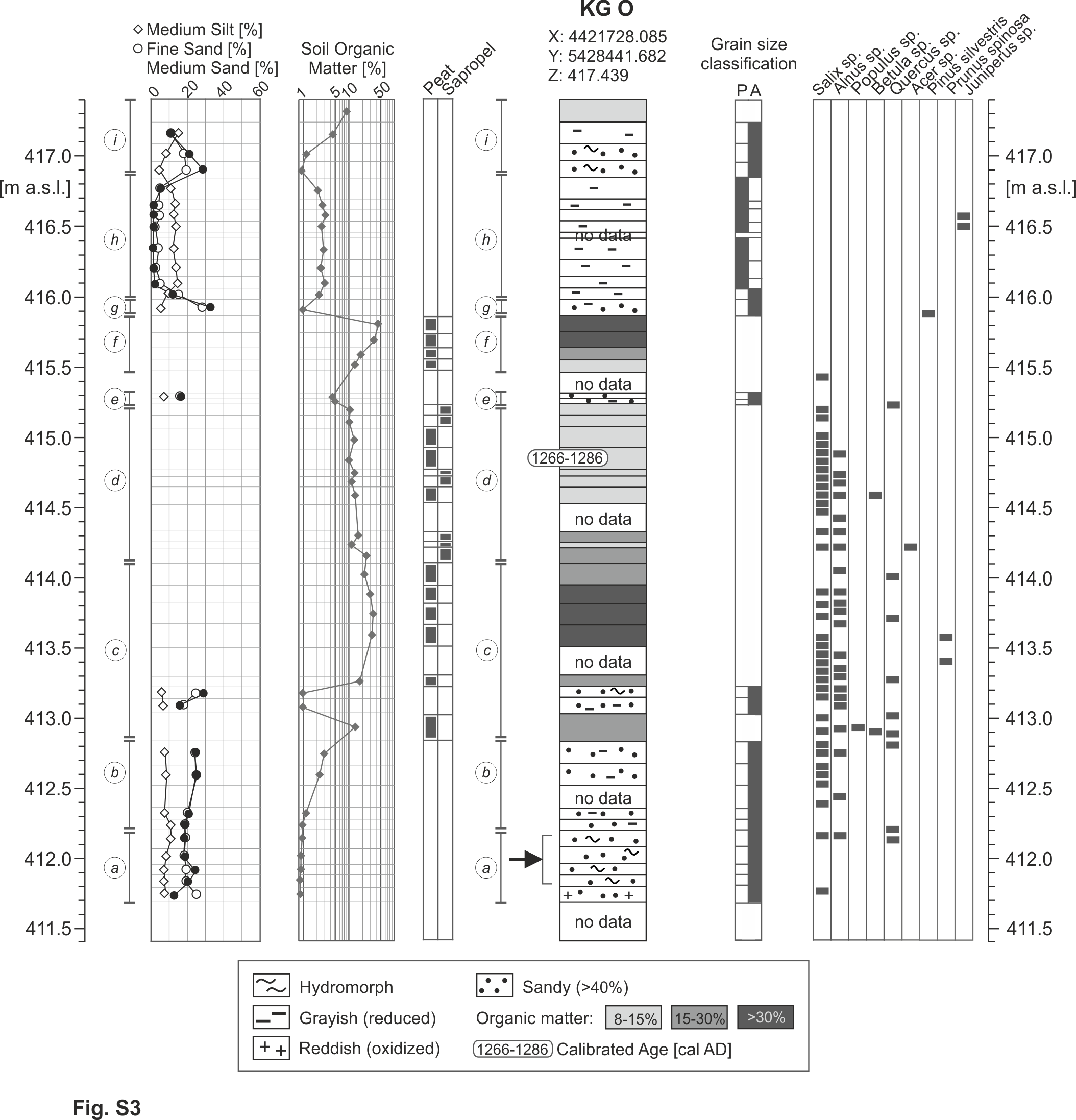

Supplement: Figure S3 — Stratigraphical documentation of core O. Anthracological findings from the West-East Section are presented in a synthetic form and normalized to the levels of core O. The black arrow marks the level of the Carolingian excavation depth. (TIF) [file pone.0108194.s003.tif]
