# Supplementary material for: Charlemagne's Summit Canal: An Early Medieval Hydro-Engineering Project for Passing the Central European Watershed
Source: PLoS One. 2014 Sep 24;9(9):e108194. doi: 10.1371/journal.pone.0108194 (PMC4177111; doi:10.1371/journal.pone.0108194)
Supplement: Table S1 — Technical and historical terminology related medieval inland navigation and canalisation. The table shows specific terms which are used in the manuscript in a stringent and standardised form. The High Medieval Period from the late 10th century to early 14th century corresponds to the Central Middle Ages following the Oxford terminology [75]. (DOCX) [file pone.0108194.s004.docx]

**Table S1**

| **Term (English)** | **Term (German)** | **Explanation** |
| --- | --- | --- |
| Bank | Wall | Excavated material of the Carolingian trench. The banks are on both sides of the trench. |
| Dam | Damm | Filled and enhanced linear structure. (barrage) |
| Embankment | Straßendamm | Filled and enhanced linear structure. (road bank) |
| Canal | Kanalbauwerk | Artificial navigable waterway |
| Trench | Graben | Carolingian linear excavation |
| Fossa Carolina | Karlsgraben | Charlemagne’s canal |
| Pond | Weiher | Artificial open water body |
| Stepped ponds | Weihertreppe | Artificial water bodies with stepped bottom surfaces |
| Summit canal | Scheitelkanal | Artificial navigable waterway with ascending and descending levels on both sides of a watershed. A hydro-engineering concept is needed to supply the summit of the canal with water. |
| Chute | Schiffsrampe | Inclined plane for towing or slipping boats up or down between two waterways of a different level |
| Weir | Stauwehr | Wooden barrage |
| Scow | Prahm | Flat-bottomed boat made for inland waterways, usually towed on a towpath |
| Hythe | Schiffslände | Landing-place or small port without a pier, characterized by landing the boat on the river bank |
| Headwater | Quellgebiet | Course of the stream close to the spring |
| Early Medieval Period | Frühmittelalter | 5^th^ to late 10^th^ century |
| High Medieval Period | Hochmittelalter | Late 10^th^ century to early 14^th^ century. This corresponds to the Central Middle Ages following the Oxford terminology. |
