# Supplementary material for: Charlemagne's Summit Canal: An Early Medieval Hydro-Engineering Project for Passing the Central European Watershed
Source: PLoS One. 2014 Sep 24;9(9):e108194. doi: 10.1371/journal.pone.0108194 (PMC4177111; doi:10.1371/journal.pone.0108194)
Supplement: File S1 — Core K and O: examples of our detailed chronostratigraphical approach. We show two representative cores from the Central Section and the West-East Section to document our stratigraphical approach in a more detailed form. (DOCX) [file pone.0108194.s005.docx]

**File S1**

**Core K in the Central Section: example of our detailed chronostratigraphical approach**

We present core K (Fig. S2) as a representative example of our detailed analytical approach which was applied for the other cores in the Central Section as well. Core K can be separated into the following sequences:

The grain sizes of the lowermost sequence (*a*) of core K (410.90 to 412.40 m, Fig. S2) are typical for the ‘fluvial type’ sediment texture (Fig. 4a). The sequence reveals a fining-up deposition process with slightly decreasing medium and fine sand contents. We interpret this sequence (*a*) as an *in situ* fluvial layer of pre-Carolingian age. The sequence is generally poor in organic matter and plant macro-remains. The transition to the lowermost Carolingian trench refills is difficult to detect. This sequence (*b*) (412.70 to 413.40 m) features also a bimodal grain size distribution with a primary maximum in medium to fine sand which is typical for the ‘fluvial type’ sediment texture. The sediment matrix is poor in organic matter as well. However, within the clastic greyish sequence numerous wood and plant remains are detectable. Three ^14^C dating from wood remains show ages between 701-773 and 813-923 cal AD (Fig. S2, Table 1) and give evidence for the Carolingian age of the sequence. Here, the ^14^C ages feature an age inversion (Fig. S2), probably induced by an abrupt re-deposition of material from the canal edges. The next sequence (*c*) (413.40 to 414.55 m) consists of a thick peat layer indicating enduring semi-terrestrial conditions within the trench. A plant remnant from the upper part of the peat layer reveals an age of 1043-1133 cal AD. Subsequently a sapropel layer between 414.55 to 414.65 m (sequence *d*) gives evidence for the existence of a former pond. The sapropel layer is covered by a second peat layer with a gradual transition to a half-bog soil (414.85 to 415.35 m, sequence *e*) indicating more terrestrial conditions. Within the second peat layer a wood remnant shows an age of 1079-1195 cal AD. The following sequence (*f*) (415.35 to 416.15 m) mirrors a ‘ponding type’ textual group (cf. Fig. 4b). We assume an open water body during the deposition phase here. In this sequence, the organic content is low in the beginning but strongly increases to the upper part. The top of core K shows a thin sand-free layer (416.15 to 416.35 m) poor in organic content.

**Core O in the West-East Section: example of our detailed chronostratigraphical approach**

We show one representative core from the West-East Section to document our stratigraphical approach in a more detailed form. Here, core O (Fig. S3) be separated into the following sequences:

The lowermost sequence (*a*) of core O (411.70 to 412.20 m, Fig. S3) features a clastic layer with two grain-size maxima in the fine to medium sandy and the fine clayey fraction. This grain size distribution is typical for the ‘fluvial type’ sediment texture (Fig. 4a). The greyish sediment matrix is poor in organic matter but reveals at the top a few plant remnants. We interpret this sequence as the transition from *in situ* fluvial deposits to the slighty plant remnant-enriched Carolingian trench bottom. The following sequence (*b*) (412.20 to 412.85 m) is characterized by a strong increase in organic matter and contents of plant macro-remnants. We postulate a first Carolingian refill of trench under semi-terrestrial conditions here. The material must be eroded from the adjacent Carolingian banks. This sequence is covered by a thick layer of weakly decomposed fen deposits (412.85 to 414.10 m, sequence *c*) with numerous wood remnants of *Salix sp*. and *Alnus sp*. The following sequence (*d*) (414.10 to 415.20 m) mirrors an alternation of semi-terrestrial peat deposits and sapropel layers. Here, we have stratigraphical evidence for the temporary existence of former ponds. A ^14^C dated wood remnant shows a High to Late Medieval age of 1266-1286 cal AD (Fig. S3, Table 2). The next sequence (*e*) (415.20 to 415.30 m) reveals a thin clastic layer with decreased organic matter. Its grain-size distribution points to the ‘fluvial type’ sediment texture (Fig. 4a) indicating a colluvial refill from the canal edges. The next sequence (*f*) (415.40 to 415.90 m) shows a gradual but strong increase in the organic matter resulting in the accumulation of a thin peat layer. We conclude a semi-terrestrial environment here. After a short but clear phase of colluvial deposition (415.90 to 416.00 m, sequence *g*) a thick sequence (*h*) (416.00 to 416.90 m) mirrors a ‘ponding type’ sediment texture. Although the clayey to silty deposits indicate only reduced contents of organic matter we assume an open water body during the deposition phase here (cf. Fig. 4b). Probably former organic matter was decomposed after the deposition of the layer due to a subsequent decrease of the groundwater table. The uppermost sequence (*i*) of core O (416.90 to 417.40 m) indicates a covering colluvial deposition of sandy bank material under an increasing impact of groundwater and the terminal development of a half-bog A horizon.
